# NLR inflammasome pathways: key targets for pathogenesis and therapy of metabolic diseases

**DOI:** 10.3389/fimmu.2026.1823724

**Published:** 2026-05-29

**Authors:** Jun Zhou, Ruohao Yang, Wanyu Zhu, Zhilin He, Yueke Ma, Zhiwei Feng, Lili Yu

**Affiliations:** 1 School of Basic Medical Sciences, Xinxiang Medical University, Xinxiang, Henan, China; 2 The Third Affiliated Hospital of Xinxiang Medical University, Xinxiang Medical University, Xinxiang, Henan, China

**Keywords:** immunometabolism, inflammasome, metabolic disease, NLR, therapeutic strategies

## Abstract

The NOD-like receptor (NLR) inflammasome system is an evolutionarily conserved intracellular surveillance network that responds to pathogen-associated molecular patterns (PAMPs), damage-associated molecular patterns (DAMPs), and metabolism-associated molecular patterns (MAMPs). Beyond the well-characterized NLRP3 inflammasome, accumulating evidence suggests that NLRP1, NLRP6, NLRC5, NLRP12, and NLRP2 are also implicated in metabolic disorders, including obesity, type 2 diabetes mellitus (T2DM), atherosclerosis, and metabolic dysfunction-associated steatotic liver disease (MASLD). This review summarizes the molecular mechanisms governing NLR inflammasome activation and discusses the divergent, context-dependent roles of selected NLR family members in metabolic inflammation. We distinguish established inflammasome-dependent pathways from emerging inflammasome-independent and PANoptosis-related mechanisms, with particular attention to species differences, disease context, and strength of evidence. Therapeutic strategies targeting inflammasome components or downstream effectors are critically evaluated, including small-molecule inhibitors, cytokine blockade, peptide-derived agents, and natural bioactive compounds. By integrating mechanistic findings with a translational evidence hierarchy spanning *in vitro* studies, animal models, human observational data, early clinical trials, randomized evidence, and approved or repurposed anti-inflammatory therapies, this review highlights both the promise and limitations of precision inflammasome modulation for metabolic disease intervention, providing an evidence-graded therapeutic perspective on NLR biology in metabolic disease.

## 1 Introduction

The global prevalence of metabolic diseases, including obesity, type 2 diabetes mellitus (T2DM),and atherosclerosis, has reached epidemic proportions and poses an increasing threat to human health. Increasing evidence indicates that chronic low-grade inflammation is a central driver in the initiation and progression of metabolic diseases (1). NOD-like receptors (NLRs) and their inflammasomes, especially NLRP3, have been implicated as key molecular mediators linking metabolic dysfunction and chronic inflammation, and emerging evidence further supports the involvement of NLRP1, NLRP6, NLRC5, and NLRP12 (2).

**Figure 1:**
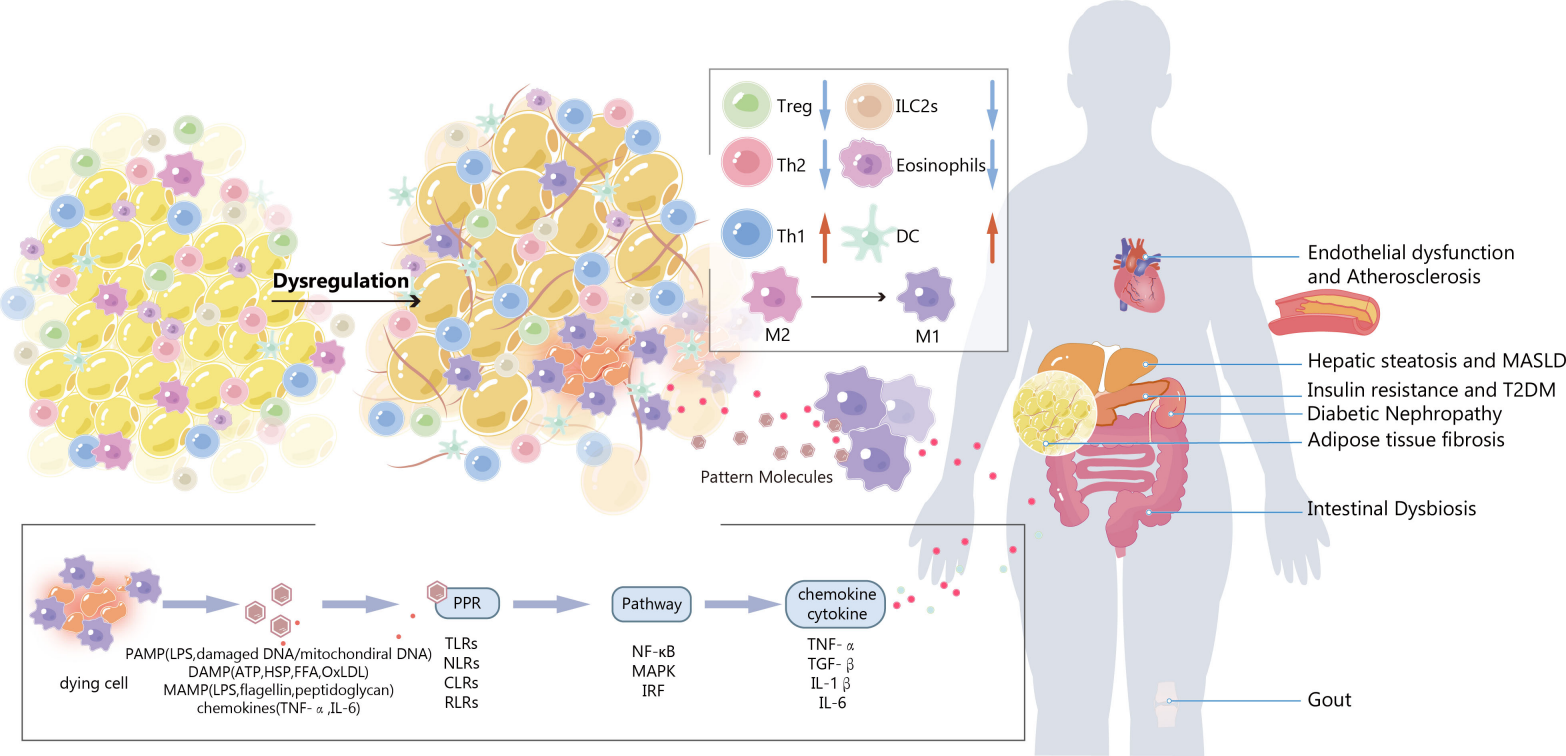
Inflammation links obesity to metabolic disease. Obesity promotes chronic low-grade inflammation through adipose tissue expansion, immune-cell remodeling, gut barrier dysfunction, and the release or accumulation of pathogen-associated molecular patterns (PAMPs), damage-associated molecular patterns (DAMPs), and metabolism-associated molecular patterns (MAMPs). These signals activate pattern-recognition receptors, including Toll-like receptors (TLRs), NOD-like receptors (NLRs), and C-type lectin receptors (CLRs), thereby activating inflammatory pathways such as NF-κB, MAPK, and interferon-related signaling. These inflammatory responses contribute to insulin resistance and metabolic complications, including MASLD/MASH, atherosclerosis, diabetic nephropathy, and other obesity-associated disorders.

The NLR family plays an indispensable role in the host immune system. As a class of evolutionarily conserved intracellular pattern recognition receptors, they can sensitively detect various danger signals, including pathogen-associated molecular patterns (PAMPs), damage-associated molecular patterns (DAMPs), and metabolism-associated molecular patterns (MAMPs) (3, 4). During infection or tissue injury, specific NLRs detect microbial ligands or endogenous danger signals. For example, NAIP5 directly recognizes flagellin, and NLRC4 acts as a downstream adaptor to assemble the inflammasome complex; NLRC4 itself does not function as a direct sensor for flagellin (5). Endogenous danger signals such as extracellular ATP can activate the NLRP3 inflammasome and promote caspase-1-dependent interleukin-1β (IL-1β) maturation (6). Activated inflammatory caspases cleave gasdermin D (GSDMD), enabling pore formation and pyroptosis-associated cytokine release (7–9). Following ligand recognition or cellular perturbation, inflammasome-forming NLRs can assemble multiprotein complexes involving apoptosis-associated speck-like protein containing a CARD (ASC) and pro-caspase-1, thereby activating caspase-1 and promoting IL-1β/interleukin-18 (IL-18) maturation (10) (**Figure 1**).

In the obese state, pathological expansion and dysfunction of adipose tissue lead to the accumulation or release of metabolism-associated danger signals. These MAMPs include free fatty acids generated by enhanced lipolysis, oxidized low-density lipoprotein, cholesterol or cholesterol crystals, glucose-derived metabolites, and advanced glycation end products advanced glycation end-products These metabolic danger signals can engage pattern recognition receptors-mediated inflammatory pathways, including nuclear factor kappa-light-chain-enhancer of activated B cells (NF-κB), mitogen-activated protein kinase (MAPK), and NLRP3 inflammasome signaling, thereby contributing to chronic low-grade inflammation and inflammatory cytokine production (4, 11).

Meanwhile, gut microbiota dysbiosis and impaired intestinal barrier function associated with obesity collectively exacerbate systemic low-grade inflammation. Changes in the gut microbiota structure result in alterations in the composition and abundance of PAMPs and MAMPs. Increased intestinal permeability due to reduced expression of tight junction proteins, such as occludin and claudin, allows microbial-derived molecules to translocate into the circulation. These molecules activate Toll-like receptors on immune cells and may indirectly contribute to NLR inflammasome activation, amplifying systemic low-grade inflammation (12).

Consequently, MAMPs released from adipose tissue and PAMPs/DAMPs translocated from the intestine together form a persistent inflammatory stimulus network. Through their synergistic effect, they drive the excessive activation of inflammasomes and trigger the cascade release of proinflammatory cytokines, including IL-1β and IL-18. This vicious cycle not only directly impairs the insulin sensitivity of peripheral tissues, inducing insulin resistance, hyperglycemia, and metabolic dysfunction-associated steatotic liver disease (MASLD), but also connects metabolic inflammation closely with β-cell dysfunction in the pathogenesis of T2DM through the resulting systemic inflammatory environment (13).

Given the pivotal role of NLRs and their inflammasomes in the pathogenesis of metabolic disorders, elucidating their activation mechanisms and crosstalk with metabolic dysregulation, along with identifying potential therapeutic targets, holds significant translational value for developing novel treatment strategies. Investigations focusing on NLRs and their inflammasomes have emerged as a major research frontier in metabolic diseases, aiming to develop novel preventive and therapeutic approaches.

### 1.1 Literature search strategy and evidence assessment

This narrative review was based on a structured literature search of PubMed, Web of Science, and Google Scholar up to April 2026. Search terms included combinations of “NOD-like receptor,” “NLR,” “inflammasome,” “NLRP3,” “NLRP1,” “NLRP6,” “NLRC5,” “NLRP12,” “NLRP2,” “PANoptosis,” “pyroptosis,” “obesity,” “type 2 diabetes,” “atherosclerosis,” “metabolic dysfunction-associated steatotic liver disease,” “MASLD,” “MASH,” “NAFLD,” “NASH,” “MAFLD,” “immunometabolism,” “insulin resistance,” and “therapeutic targeting.” Additional searches were performed for therapeutic terms, including “NLRP3 inhibitor,” “IL-1β blockade,” “IL-1 receptor antagonist,” “small-molecule inhibitor,” “natural product,” and “bioactive compound.” Because liver-disease terminology has changed over time, studies using earlier terms such as NAFLD, NASH, or MAFLD were considered when their disease context corresponded to the current MASLD/MASH framework.

Priority was given to original mechanistic studies, animal models with metabolic phenotyping, human tissue or observational studies, clinical trials, and authoritative consensus or nomenclature statements. Review articles were used primarily to provide background context and to identify additional primary literature. Studies were evaluated qualitatively according to experimental system, disease relevance, reproducibility, and translational strength. Evidence was considered stronger when supported by human data together with animal or mechanistic studies, moderate when mainly supported by animal and mechanistic evidence with limited human association data, and limited or emerging when based primarily on cell models, mouse studies, epigenetic associations, or hypothesis-generating findings. Findings related to NLRC5, NLRP12, PANoptosis, and inflammasome-independent functions were therefore interpreted cautiously, with particular attention to species differences, tissue specificity, and whether the mechanism has been validated in human metabolic disease.

## 2 NLRs and inflammasome pathways

**Figure 2:**
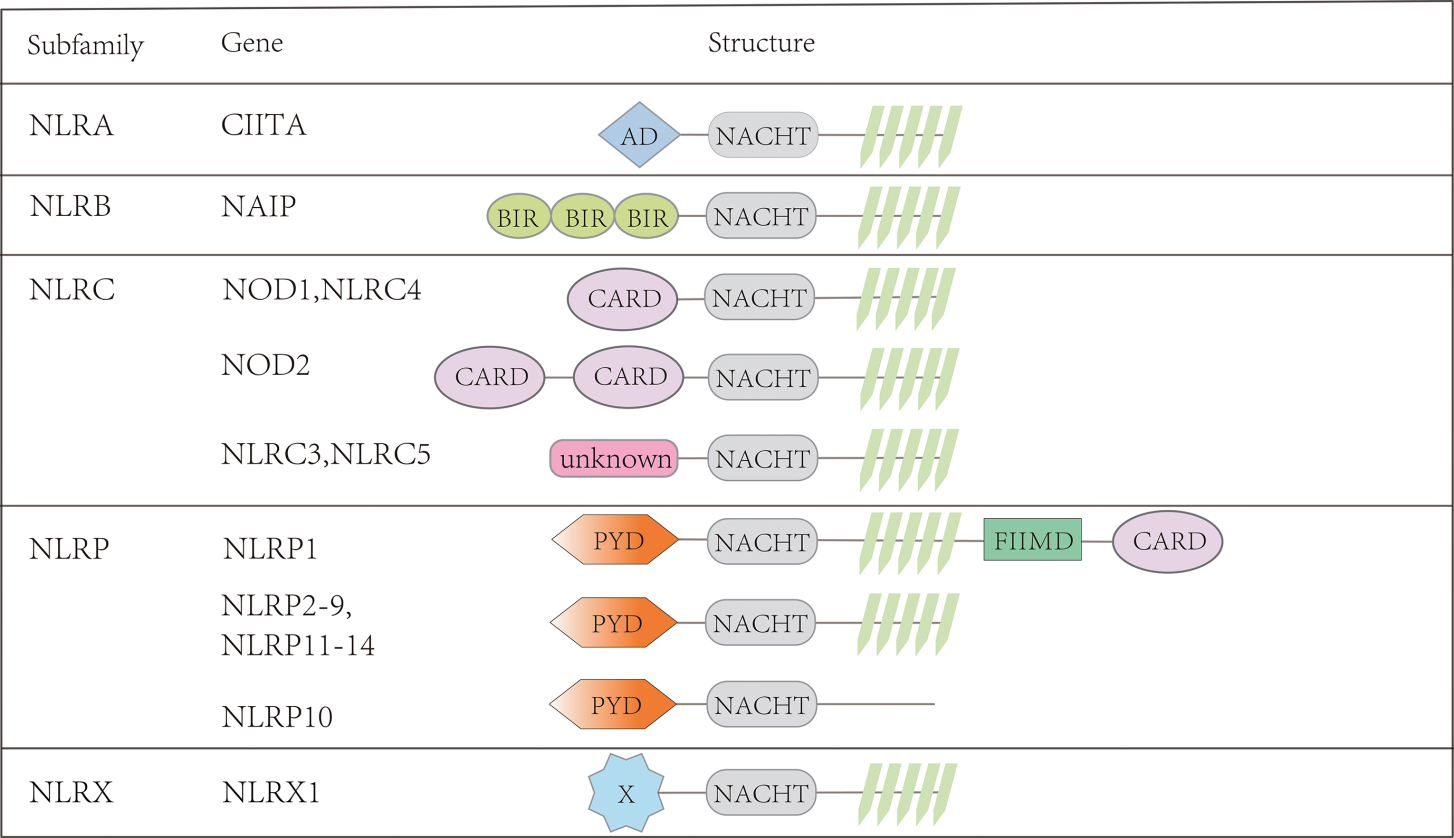
The NOD-like receptor family. Schematic representation of NOD-like receptor (NLR) subfamilies and their domain organization. NLR proteins typically contain a central NACHT domain, C-terminal leucine-rich repeats (LRRs), and variable N-terminal effector domains, including pyrin domains (PYDs), caspase recruitment domains (CARDs), acidic transactivation domains, or baculovirus inhibitor of apoptosis protein repeat domains.

NLRs are a family of cytosolic innate immune receptors characterized by their NOD domain. As intracellular pattern recognition receptors, NLRs play pivotal roles in host defense and homeostasis maintenance through diverse immunological functions. The NLR family comprises 23 distinct members in humans and at least 34 NLR genes in mice, which can be functionally categorized into inflammasome-forming and non-inflammasome-forming subfamilies based on their structural domains and downstream signaling capabilities (14, 15). Canonical NLRs possess three functional domains: a C-terminal leucine-rich repeat (LRR) domain for ligand recognition, a central NACHT domain that shares homology with NAIP, CIITA, HET-E, and TP1 proteins and is responsible for nucleotide-dependent oligomerization, and an N-terminal effector domain that recruits downstream signaling components (16) (**Figure 2**). The NACHT and LRR domains are evolutionarily conserved structural modules in many NLRs. The LRR domain contributes to ligand sensing and autoregulation, although direct binding to PAMPs or DAMPs has been established only for selected NLRs. Ligand recognition or cellular perturbation can induce conformational changes that relieve autoinhibition and promote NACHT-dependent oligomerization. This domain, comprising seven conserved segments including an ATP/GTPase-specific P-loop motif, a Mg^2+^-binding site, and five additional signature motifs, serves as the molecular engine that drives NTPase activity and oligomerization. The N-terminal effector domain consists of either a caspase recruitment domain (CARD) or a pyrin domain (PYD), which function as molecular scaffolds to bridge NLR receptors with downstream adaptor proteins and effector molecules, thereby initiating signal transduction cascades. Some NLRs alternatively carry a baculovirus inhibitor of apoptosis protein repeat domain at their N-terminus. These effector domains initiate signal transduction by engaging in death domain superfamily interactions with proteins such as caspases and inhibitor of apoptosis family members, with their functional output being inflammasome assembly.

NLRs are divided into five subfamilies based on their variable N-terminal domains, including NLRA (containing acidic transactivation domains), NLRB (bearing BIR domains), NLRC (featuring CARD domains), NLRX (with unknown effector domains), and NLRP (possessing PYD). The NLRP subfamily is the largest group of NLRs, including 14 human members (NLRP1-14) that detect both pathogen-derived and sterile danger signals. After N-terminal conformational changes, NLRPs activate the adapter protein ASC through homotypic PYD-PYD or CARD-CARD interactions, which then initiates inflammasome complex assembly (17, 18). ASC contains both a PYD and a CARD, enabling its interaction with diverse sensor proteins. The resulting sensor-ASC oligomeric complex recruits procaspase-1 through CARD-CARD interactions, initiating inflammasome assembly signaling (19). This recruitment induces autocatalytic cleavage and activation of procaspase-1. The mature caspase-1 then proteolytically cleaves its substrates, including the pore-forming protein GSDMD, producing C-terminal and N-terminal fragments that carry out distinct biological functions (7). Concurrently, caspase-1 cleaves the proforms of IL-1β and IL-18 (pro-IL-1β and pro-IL-18), generating biologically active mature IL-1β and IL-18 (20, 21). The N-terminal GSDMD fragment translocates to the plasma membrane, where it oligomerizes to form functional pores. Concurrently, NINJ1 is recruited to these sites, mediating plasma membrane rupture through a complementary mechanism (22). The formation of transmembrane pores and subsequent plasma membrane rupture induce osmotic cell lysis, resulting in the release of cytokines such as IL-1β and IL-18 and DAMPs (8, 9). This lytic, proinflammatory cell death process is defined as pyroptosis. NLRPs do not mediate transcriptional activation of inflammatory mediators but instead orchestrate pyroptotic cell death by serving as essential components of inflammasomes that regulate caspase-1 activation. Additionally, activation of caspase-11 (in mice) or caspases-4/-5 (in humans) can drive pyroptosis through the non-canonical inflammasome pathway.

Recent studies have proposed that selected NLRs, including NLRP12 and NLRC5, may participate in PANoptosis-related cell death complexes under specific infectious or metabolic-stress conditions. PANoptosis is a lytic inflammatory cell death pathway mediated by PANoptosome complexes that integrate molecular features of pyroptosis, apoptosis, and necroptosis through caspases, receptor-interacting serine/threonine kinases (RIPKs), and gasdermin family proteins (23, 24). However, the relevance of PANoptosis to metabolic disease remains an emerging area, and its generalizability across tissues, species, and human disease settings has not been established. Therefore, PANoptosis is discussed here as a hypothesis-generating mechanism rather than a consensus pathway. In selected experimental models, NLRP12 has been proposed to contribute to PANoptosome assembly through interactions with cell death effectors such as caspase-8, RIPK3, and GSDMD, thereby potentially coordinating crosstalk among distinct cell death pathways (25). In obesity-related models, hematopoietic cell kinase has been reported to bind the NACHT-PYD interface of NLRP12 and promote PANoptosome assembly, which may contribute to adipose tissue inflammation and insulin resistance (25). Similarly, NLRC5 has recently been proposed to act as an intracellular sensor of NAD+ depletion under obesity-associated metabolic stress, such as high-fat diet exposure. In this context, NLRC5 may participate in a PANoptosome complex involving NLRP12, ASC, and RIPK3 and may coordinate activation of GSDMD-mediated pyroptosis, caspase-3/7-mediated apoptosis, and mixed lineage kinase domain-like proteinmediated necroptosis, thereby contributing to adipose tissue inflammation and hepatic injury in selected experimental models (26).

## 3 Selected NLR family members in metabolic diseases

The NLR inflammasome system is an evolutionarily conserved intracellular surveillance network that detects cellular perturbations through the recognition of PAMPs, DAMPs, and MAMPs. Dysregulation of NLR inflammasomes has been implicated in metabolic and inflammatory disorders, including obesity, T2DM and its complications, MASLD, and atherosclerosis.

Among the 23 known human NLRs, this review focuses on NLRP3, NLRP1, NLRP6, NLRC5, NLRP12, and NLRP2 because these members have been most frequently implicated in obesity-related low-grade inflammation, diabetes, atherosclerosis, and metabolic liver disease. NLRP3 and NLRP1 are discussed as relatively well-characterized inflammasome-forming NLRs. NLRP6 and NLRP12 are included because they have been reported to exert both inflammasome-dependent and inflammasome-independent functions in a context-dependent manner. NLRC5 is included primarily because of its immunometabolic relevance and its best-established role as a transcriptional regulator of major histocompatibility complex class I (MHC-I) genes, rather than as a canonical inflammasome sensor (27, 28). NLRP2 is included as an emerging regulator with limited but relevant evidence in metabolic liver disease. By contrast, NLRC4, NOD1, and NOD2 are briefly mentioned but not reviewed in equal depth. NLRC4 is mainly involved in NAIP-dependent bacterial ligand sensing and inflammasome activation (5), whereas NOD1 and NOD2 primarily signal through RIPK2-mediated NF-κB and MAPK pathways rather than forming classical NLRP-type inflammasomes. Therefore, these members fall outside the main therapeutic and evidence-graded focus of this review.

### 3.1 NLRP3 inflammasome

**Figure 3:**
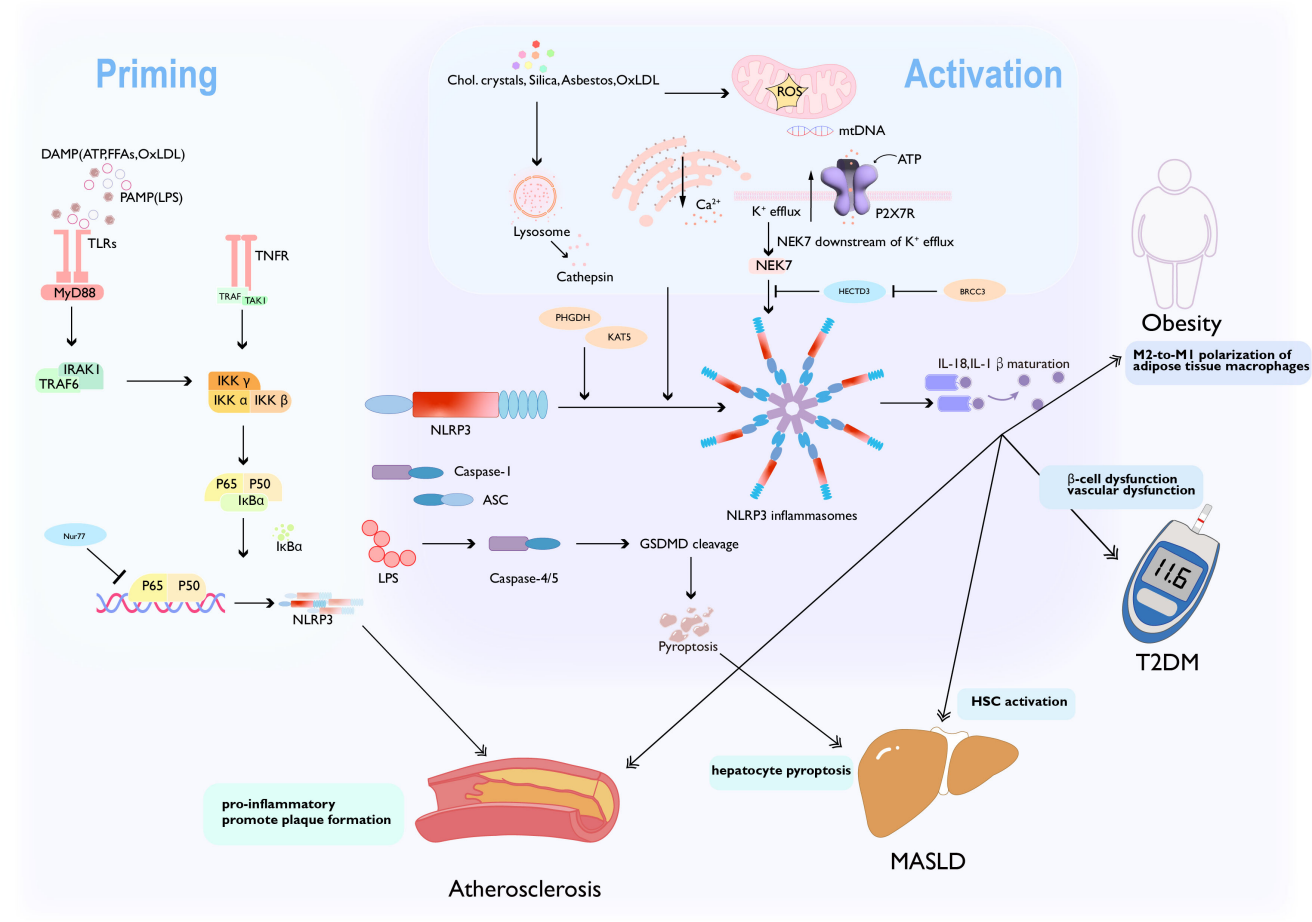
NLRP3 inflammasome activation in metabolic disorders. NLRP3 inflammasome activation involves two major steps: priming and activation. During priming, TLRs and TNFR activate NF-κB signaling, promoting transcription of NLRP3 and pro-inflammatory mediators. During activation, metabolic and cellular stress signals, including cholesterol crystals, oxLDL, extracellular ATP, ROS, lysosomal cathepsin release, mitochondrial dysfunction, and K^+^ efflux, drive NLRP3 inflammasome assembly. NEK7 acts downstream of K^+^ efflux to facilitate NLRP3 activation. Activated NLRP3 recruits ASC and pro-caspase-1, leading to caspase-1 activation, IL-1β/IL-18 maturation, gasdermin D cleavage, and pyroptosis. NLRP3 inflammasome activation drives chronic inflammation, contributing to the pathogenesis of obesity, T2DM, MASLD, and atherosclerosis.

The NLRP3 inflammasome has emerged as one of the most extensively studied inflammasomes, capable of responding to a wide range of intracellular and extracellular danger signals to initiate inflammatory responses. Its activation is strongly implicated in the pathogenesis of various metabolic disorders (**Figure 3**).

#### 3.1.1 Activation of the NLRP3 inflammasome

The NLRP3 inflammasome is a central intracellular sensor of cellular stress that responds to a broad range of microbial, endogenous, and metabolic stimuli. Rather than directly recognizing a single conserved ligand, NLRP3 is generally activated by cellular perturbations such as ion flux, mitochondrial dysfunction, lysosomal damage, and metabolic stress (29, 30). NLRP3 activation is commonly described as a two-step process consisting of priming and activation (31). During the priming step, Toll-like receptors (TLRs) and cytokine receptors, including TNF receptor and IL-1 receptor, activate NF-κB signaling and thereby increase the transcriptional expression of NLRP3, pro-IL-1β, and, in some contexts, pro-IL-18 (31). During the activation step, diverse exogenous and endogenous stimuli, including extracellular ATP, pore-forming toxins, crystalline or particulate matter, and metabolic danger signals, disrupt intracellular homeostasis (29). These stimuli promote NLRP3 inflammasome assembly through mechanisms that include K^+^ efflux, Ca^2+^ signaling, mitochondrial dysfunction, lysosomal rupture, reactive oxygen species (ROS) generation, and NIMA-related kinase 7 (NEK7)-dependent conformational licensing of NLRP3 (30). The activated NLRP3 inflammasome recruits ASC and pro-caspase-1 to form the NLRP3–ASC–pro-caspase-1 complex, leading to caspase-1 activation, IL-1β and IL-18 maturation, and gasdermin D-mediated pyroptosis (7). The priming and activation model is well established for NLRP3, but the relative contribution of each signal varies among metabolic stimuli and cell types; therefore, stimulus-specific mechanisms are described separately below.

Post-translational modifications play important roles in regulating the canonical NLRP3 inflammasome pathway, particularly during the activation phase by controlling NLRP3 conformational changes and inflammasome assembly. For instance, acetylation at lysine 24 is critical for NLRP3 oligomerization, with lysine acetyltransferase 5 (KAT5) mediating this modification. Genetic deficiency or pharmacological inhibition of KAT5 significantly attenuates inflammasome activation (32). Similarly, phosphoglycerate dehydrogenase (PHGDH), the rate-limiting enzyme in serine biosynthesis, promotes NLRP3 inflammasome activity by facilitating acetylation of pyrin domain residues K21/22/24, which may suppress ubiquitination of both NLRP3 and ASC. The role of PHGDH appears to involve elevation of acetyl-CoA through the serine-one-carbon metabolic axis, thereby providing substrate for KAT5. Concurrently, PHGDH inhibits the activity of the deacetylase sirtuin 2, which reverses NLRP3 acetylation, thereby promoting enhanced acetylation and attenuated deacetylation (33). Beyond direct Post-translational modifications, several NLRP3-interacting regulatory proteins also influence inflammasome assembly. The E3 ubiquitin ligase HECTD3 suppresses inflammasome assembly in an E3 activity-independent manner by competitively disrupting the NLRP3–NEK7 interaction through direct binding of its DOC domain to the NACHT/LRR domains of NLRP3 (34). In the non-canonical pathway, murine caspase-11 and human caspase-4/5 directly sense cytosolic lipopolysaccharide (LPS) and cleave GSDMD. The resulting GSDMD pores induce K^+^ efflux and other secondary signals that subsequently promote NLRP3 inflammasome activation (35).

#### 3.1.2 NLRP3 inflammasome in obesity

The NLRP3 inflammasome plays a pivotal role in the pathogenesis of obesity and related metabolic disorders by driving disease progression through its dual promotion of chronic low-grade inflammation and systemic metabolic dysfunction. In the obese state, adipose tissue undergoes pathological expansion characterized by adipocyte hypertrophy, resulting in localized hypoxia and cellular stress that triggers activation of both adipocytes and resident immune cells, such as macrophages. This proinflammatory microenvironment stimulates the robust release of DAMPs, including high mobility group box 1, heat shock proteins, and crystalline urate, as well as fragmented extracellular matrix components such as hyaluronan fragments. It also induces the production of MAMPs, including free fatty acids (FFAs) and oxidized low-density lipoprotein (36, 37).

The classification of saturated fatty acids such as palmitic acid as direct TLR4 ligands remains controversial. Accumulated evidence indicates that TLR4 requires the accessory protein myeloid differentiation factor 2 for efficient ligand recognition and downstream signal transduction (38). Palmitic acid does not interact directly with TLR4, but instead binds to the hydrophobic pocket of myeloid differentiation factor 2 to initiate TLR4 signaling indirectly under conditions of elevated FFAs and high-fat diet-related tissue injury (39). This indirect and context-dependent regulatory mechanism has also been confirmed in human monocyte-derived dendritic cells (40). Upon activation, the MyD88–NF-κB signaling axis is triggered to upregulate NLRP3 and pro-IL-1β expression, providing essential molecular components for subsequent inflammasome assembly. FFAs also induce endoplasmic reticulum stress and mitochondrial dysfunction, generating ROS that serve as critical secondary messengers to facilitate NLRP3-NEK7 interaction and subsequent inflammasome assembly. Recent investigations have identified upregulated expression of the TRPM7 cation channel in adipose tissue of obese murine models, where it potentiates FFA-induced inflammatory responses through calcium-dependent activation of the TAK1-NF-κB signaling cascade (41). Emerging evidence establishes the fatty acid biosynthesis pathway as a central mechanism governing NLRP3 inflammasome activation, mediated through fatty acid synthase-dependent palmitoylation of NLRP3 at cysteine 898, a post-translational modification essential for inflammasome complex assembly. Palmitoylation of NLRP3 facilitates its translocation to the Golgi apparatus, where it initiates inflammasome assembly. Pharmacological inhibition of either fatty acid synthase or the palmitoylation process itself significantly attenuates caspase-1 activation and IL-1β production, demonstrating the critical regulatory role of this post-translational modification in inflammasome activation (42).

In addition to fatty acids, cholesterol crystals, a well-characterized class of DAMPs, have been established as potent NLRP3 activators within atherosclerotic plaques. This activation mechanism, involving macrophage phagocytosis-induced lysosomal membrane destabilization and subsequent cathepsin B release, may similarly contribute to NLRP3 inflammasome assembly in obese adipose tissue. In obesity, necrotic adipocytes release substantial amounts of ATP, which functions as a DAMP by activating P2X7 receptors to induce potassium efflux and pannexin-1 channel opening, which are critical steps in NLRP3 inflammasome activation. Concurrently, mitochondrial damage releases mitochondrial DNA and cardiolipin that directly interact with NLRP3 to promote its oligomerization (43).

The NLRP3 inflammasome plays a key role in macrophage activation and infiltration. Specifically, it promotes the mature secretion of IL-1β and IL-18, thereby driving the migration of macrophages to inflammatory sites and inducing their polarization toward the M1 phenotype—an effect that further amplifies the local inflammatory response. Relevant studies have provided direct evidence for this regulatory axis, demonstrating that SNX10-mediated activation of the NLRP3 inflammasome enhances M1 polarization of macrophages. This enhanced M1 polarization, in turn, exacerbates tissue damage by perpetuating pro-inflammatory signaling cascades and disrupting tissue homeostasis (44). NLRP3 lacks transcription factor activity and should not be described as directly binding the Lcn2 promoter. Available evidence suggests that NLRP3 can influence Lcn2 expression through an inflammasome-independent regulatory mechanism, but the transcriptional intermediates and cell-type specificity require further clarification. Therefore, the TNFα–NLRP3–Lcn2 axis should be interpreted as a proposed regulatory pathway linking adipocyte dysfunction and macrophage activation rather than as a fully established transcriptional mechanism (45, 46). Studies have shown that caloric restriction suppresses the expression of the matricellular protein secreted protein acidic and rich in cysteine (SPARC) in adipose tissue. Mechanistically, SPARC activates the NLRP3 inflammasome during the priming phase, while its downregulation attenuates macrophage-mediated inflammation in adipose tissue. Conversely, excessive SPARC promotes macrophage activation via the JNK signaling pathway (47). Both caloric restriction and exercise-induced weight loss reduce NLRP3 expression in adipose tissue of obese individuals with T2DM, thereby attenuating inflammation and improving insulin sensitivity (48).

Overall, NLRP3 has the strongest evidence base among NLRs in obesity, with support from human adipose tissue studies, animal models, and intervention studies showing reduced inflammatory markers after weight loss. Nevertheless, most mechanistic details derive from macrophage- or adipocyte-centered experimental systems, and the causal contribution of specific metabolic triggers in humans remains incompletely defined. Therapeutically, NLRP3 inhibition is promising but still requires careful evaluation of tissue specificity, immune-suppression risk, and long-term metabolic safety.

#### 3.1.3 NLRP3 inflammasome in MASLD/MASH

MASLD, formerly known as NAFLD and overlapping with the earlier MAFLD nomenclature adopted in some studies, is defined by excessive hepatic steatosis driven by metabolic dysfunction. Its progressive inflammatory subtype is metabolic dysfunction-associated steatohepatitis (MASH), formerly referred to as NASH. Its inflammatory and progressive form is now termed metabolic dysfunction-associated steatohepatitis (MASH; previously NASH). The pathogenesis of MASLD involves complex interactions among hepatocytes, Kupffer cells, hepatic stellate cells, infiltrating immune cells, and metabolic stress signals, with NLRP3 inflammasome activation serving as an important link between metabolic dysfunction, hepatic inflammation, and fibrosis. Notably, transcriptomic analyses have revealed significant upregulation of NLRP3 inflammasome components, including NLRP3, IL-1β, and IL-18, in hepatic tissues of obese individuals with T2DM, suggesting that sustained inflammasome activation may contribute to diabetes-associated hepatic metabolic dysfunction (49). During progression from MASLD to MASH, increased hepatocyte caspase-1 activity has been reported, suggesting an association between pyroptotic activity and disease severity (50).

Studies have revealed cell-type-specific expression patterns and functional heterogeneity of NLRP3 across different hepatic cell populations. NLRP3-mediated hepatocyte pyroptosis represents one of its core pathological mechanisms. In primary hepatocytes, metabolic stressors such as free fatty acids and cholesterol crystals induce NLRP3 inflammasome assembly through CMPK2-mediated mitochondrial dysfunction (characterized by excessive mitochondrial DNA synthesis) and lysosomal rupture, ultimately triggering caspase-1-dependent pyroptosis. The inflammasome protein complexes (containing ASC specks) released by pyroptotic cells are internalized by neighboring hepatic stellate cells, where they activate TLR4/MyD88 signaling to drive hepatic stellate cells activation (upregulated α-SMA) and IL-1β secretion, thereby establishing a pro-fibrotic microenvironment (51).

Within the MASH microenvironment, lipid droplets released by damaged hepatocytes drive the differentiation of TREM2^+^ MASH-associated macrophages, previously described as NASH-associated macrophages in the original literature. This distinct subset facilitates NLRP3 inflammasome assembly via direct MS4A7 protein binding, amplifying IL-1β/IL-18 secretion. Genetic ablation of MS4A7 markedly attenuates liver injury in murine models, establishing this axis as a pathogenic mechanism in MASH progression (52). A separate study demonstrates that Zbtb18 suppresses NLRP3 inflammasome activity by modulating FXR-mediated fatty acid oxidation and transcriptionally activating CLTC protein. Under cholestatic conditions, cholangiocytes modulate NLRP3 expression through the FXR/Zbtb18 axis. Hepatic Zbtb18 deletion reduces fatty acid oxidation while enhancing CLTC-mediated NLRP3 endocytosis, thereby accelerating MASLD progression. These findings reveal a novel regulatory mechanism and provide potential therapeutic targets for NLRP3-driven liver diseases such as MASLD (53).

#### 3.1.4 NLRP3 inflammasome in atherosclerosis

Activation of the NLRP3 inflammasome serves as a pivotal driver of disease progression in atherosclerosis. In atherosclerotic plaques, phagocytic uptake of calcium phosphate and cholesterol crystals induces lysosomal destabilization and rupture, resulting in cathepsin B release from lysosomes, which subsequently triggers NLRP3 inflammasome activation. Activated NLRP3 inflammasome mediates IL-1β secretion, which amplifies inflammatory responses by promoting the activation and proliferation of monocytes, macrophages, endothelial cells, and vascular smooth muscle cells, thereby accelerating atherosclerosis progression (35). Clinical pathological studies provide direct evidence for crystal-induced inflammasome activation. Analysis of carotid endarterectomy specimens revealed a 3-fold enrichment of NLRP3-positive extracellular vesicles in atherosclerotic plaques compared to adjacent normal vessel walls, with predominant localization to macrophages surrounding crystalline deposits (54). These plaque-derived extracellular vesicles disrupt endothelial barrier integrity, creating a localized inflammatory amplifier. In patients with diabetic atherosclerosis, TLR2 exacerbates this process by dual activation of the NLRP3 inflammasome and MyD88/NF-κB signaling pathways (55).

Clonal hematopoiesis has emerged as a pivotal discovery in cardiovascular research, characterized by the selective expansion of hematopoietic stem cell clones carrying acquired somatic mutations. This phenomenon is strongly associated with increased cardiovascular disease risk. Key mechanisms involve clonal hematopoiesis, mutations in the Ten-eleven translocation 2 (TET2) gene, NLRP3 inflammasome activation, IL-1β secretion, and increased cardiovascular disease risk. TET2 deficiency potentiates NLRP3 inflammasome activation and IL-1β secretion through BRCC3-mediated NLRP3 deubiquitination. Specifically, macrophages with TET2 deficiency exhibit upregulated expression of the deubiquitinase BRCC3, which removes ubiquitin chains from the NLRP3 protein, thereby priming it for activation. This process operates independently of canonical NLRP3 activation signals such as crystalline structures or ATP, representing a sustained inflammasome activation state (56). Macrophages with TET2 deficiency demonstrate upregulated expression of pro-inflammatory genes such as IL-1β and NLRP3, revealing potential therapeutic targets for atherosclerosis intervention (57).

#### 3.1.5 NLRP3 in T2DM

T2DM, the most prevalent form of diabetes accounting for 90–95% of all cases, is characterized by chronic low-grade inflammation. Recent years have witnessed significant advances in understanding the mechanistic links between NLRP3 inflammasome activation and T2DM pathogenesis. The NLRP3 inflammasome serves as a pivotal inflammatory regulator, playing a central role in the pathogenesis and progression of T2DM. Compelling evidence demonstrates that NLRP3 inflammasome activation contributes not only to insulin resistance and β-cell dysfunction but also plays a pathogenic role in diabetic complications, including nephropathy, retinopathy, and neuropathy (58). IL-1β contributes to the pathogenesis of diabetes mellitus by inducing systemic insulin resistance through impairment of glucose uptake in key insulin-responsive tissues, including skeletal muscle, hepatic tissue, and adipose depots.

Regarding pancreatic β-cell dysfunction in T2DM patients, studies demonstrate that trimethylamine N-oxide, a gut microbiota-derived metabolite at pathological concentrations, directly impairs glucose-stimulated insulin secretion in both MIN6 murine β-cell lines and primary mouse islets. The underlying mechanism involves trimethylamine N-oxide induced upregulation of NLRP3 inflammasome-associated cytokines and depletion of sarco/endoplasmic reticulum calcium ATPase 2, which collectively suppress cytosolic calcium transients and ultimately impair insulin secretion (59).

Beyond its role in pancreatic β-cell dysfunction and impaired insulin secretion, NLRP3 inflammasome activation significantly contributes to diabetes-associated cardiovascular complications, including vascular aging and endothelial progenitor cell dysfunction. Studies demonstrate significant activation of the TLR4-NF-κB-NLRP3 signaling axis in diabetic immunosenescence, accompanied by upregulated NLRP3 gene expression. Studies utilizing NLRP3-knockout mouse models demonstrate that NLRP3 deficiency attenuates diabetes-induced vascular cell senescence mediated by immune cells, while indirectly improving vascular homeostasis through amelioration of perivascular adipose tissue dysfunction. Eight-week treatment with rapamycin and the NLRP3 inhibitor OLT1177 significantly reduced senescence markers in immune organs and improved vascular aging parameters in diabetic mice (60).

### 3.2 NLRP1 inflammasome

NLRP1 (also designated NALP1, NAC, DEFCAP, CLR17.1, or CARD7) represents the first comprehensively characterized inflammasome (61). Distinct from other NLR family members, NLRP1 possesses a unique C-terminal architecture featuring a function-to-find domain composed of ZU5 and UPA subdomains that undergoes autoproteolytic processing between these domains (62). NLRP1 mediates inflammatory responses through inflammasome assembly, a process requiring multiprotein interactions, including ASC recruitment and pro-caspase-1 binding. Subsequent caspase-1 activation drives proteolytic maturation of proinflammatory cytokines such as IL-1β and IL-18. NLRP1 can also be activated through viral double-stranded RNA (dsRNA) recognition (63). Studies have demonstrated that NLRP1 plays a regulatory role in obesity and diabetes.

#### 3.2.1 NLRP1 inflammasome in obesity

As an innate immune sensor, NLRP1 produces IL-18 during metabolic stress such as excessive energy intake, which paradoxically protects against obesity and metabolic syndrome through negative feedback regulation (64). Research demonstrates that NLRP1-dependent IL-18 production has been reported to protect against obesity and metabolic syndrome in experimental models, resembling the metabolic phenotype observed in IL-18-deficient mice. This effect is particularly pronounced in insulin-responsive tissues, including adipose tissue, skeletal muscle, and liver (64, 65).

**Figure 4:**
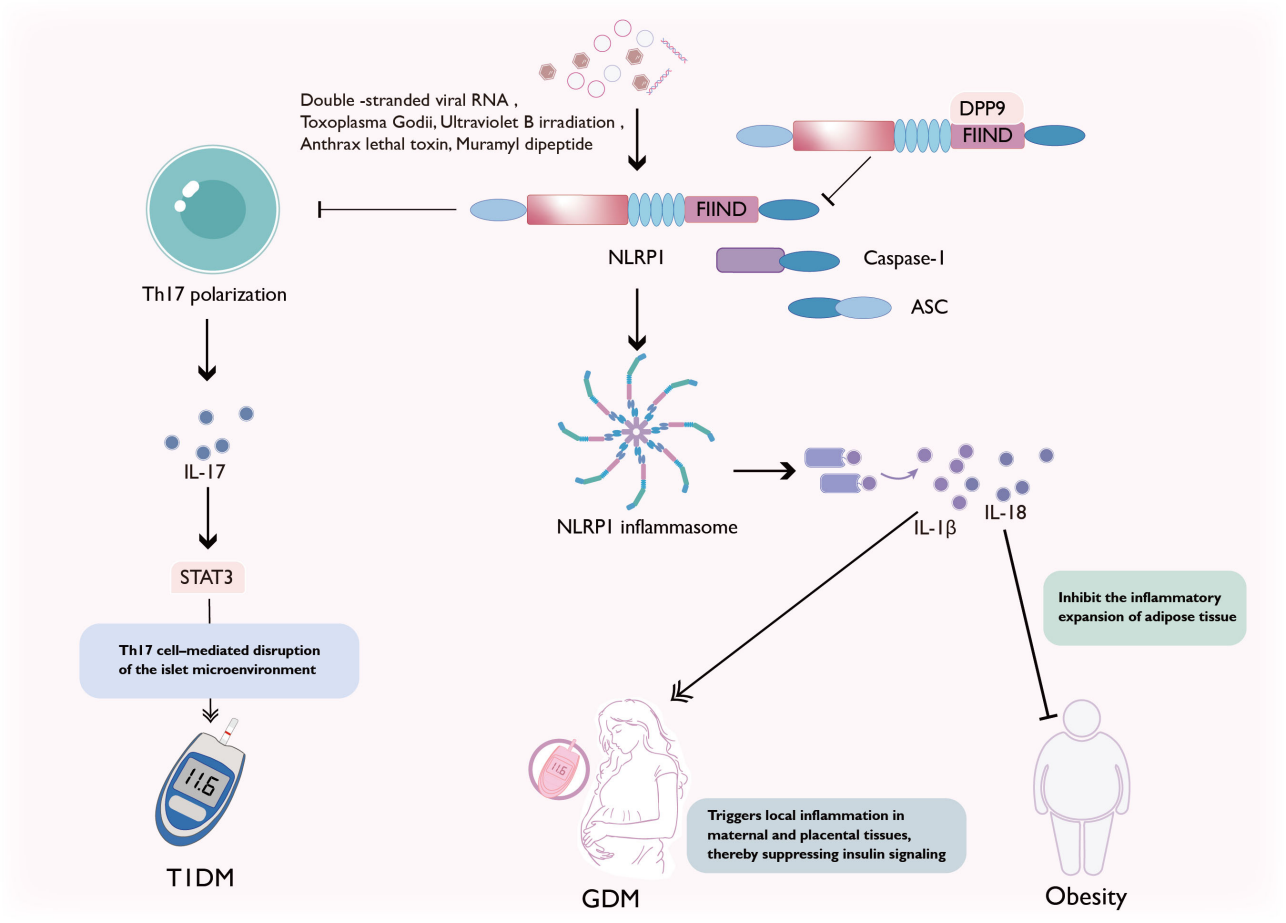
Disease-related mechanisms of NLRP1. NLRP1 inflammasome assembly is triggered by diverse stimuli, leading to recruitment of ASC and pro-caspase-1, caspase-1 activation, and maturation of IL-1β and IL-18. NLRP1 participates in obesity- and diabetes-related immune regulation: IL-18 inhibits adipose tissue inflammatory expansion, while IL-1β drives local inflammation in gestational diabetes mellitus, and Th17 polarization with subsequent IL-17 production contributes to type 1 diabetes mellitus (T1DM). The DPP9–NLRP1 axis negatively regulates inflammasome activation.

Emerging evidence suggests NLRP1 may contribute to obesity-induced chronic inflammation, and NLRP1 interacts with regulatory proteins such as CARD8 to modulate inflammasome assembly and activation (66). Moreover, studies have further identified dipeptidyl peptidase 9 (DPP9) as an interacting partner of NLRP1, suggesting additional regulatory mechanisms in inflammasome control (67). DPP9 serves as a critical regulator of inflammasome activity by suppressing NLRP1 and CARD8 activation, functioning as a key inhibitor of caspase-1-dependent pyroptosis in monocytes and macrophages (**Figure 4**). Dysregulation of this mechanism may contribute to NLRP1-associated pathologies. These findings provide novel insights into the role of NLRP1 in obesity and inflammation. While direct studies on NLRP1-obesity relationships remain limited, investigations of DPP9-NLRP1 interactions offer valuable mechanistic clues.

#### 3.2.2 NLRP1 inflammasome in diabetes-related immune dysregulation

Beyond metabolic regulation, NLRP1 functions as a negative regulator of T cell differentiation. Downregulation of NLRP1 expression markedly enhances Th17 cell polarization and IL-17 production, amplifying inflammatory responses through STAT3-dependent signaling. In murine models, NLRP1 deficiency accelerates streptozotocin-induced diabetes progression (65). Clinically, patients with type 1 diabetes mellitus (T1DM) carrying the NLRP1 SNP (rs12150220) display aberrant IL-17 expression, further supporting its immunomodulatory role in T1DM pathogenesis. These findings demonstrate that NLRP1 plays a pivotal role in maintaining T cell immune tolerance and preventing pancreatic islet autoimmunity, highlighting its potential as a therapeutic target for early-stage T1DM intervention (68).

### 3.3 NLRP6 inflammasome

NLRP6 (also known as NALP6 or PYPAF5) is a crucial member of the NLR family that functions as a cytosolic sensor for PAMPs and DAMPs. It is highly expressed in specialized epithelial cells such as intestinal epithelial cells and goblet cells in both humans and mice, with more modest expression detected in the liver, brain, kidney, and lung. Structurally, NLRP6 contains an N-terminal PYD that mediates inflammasome assembly, a central NACHT domain responsible for oligomerization, and a C-terminal LRR domain implicated in potential ligand recognition.

**Figure 5:**
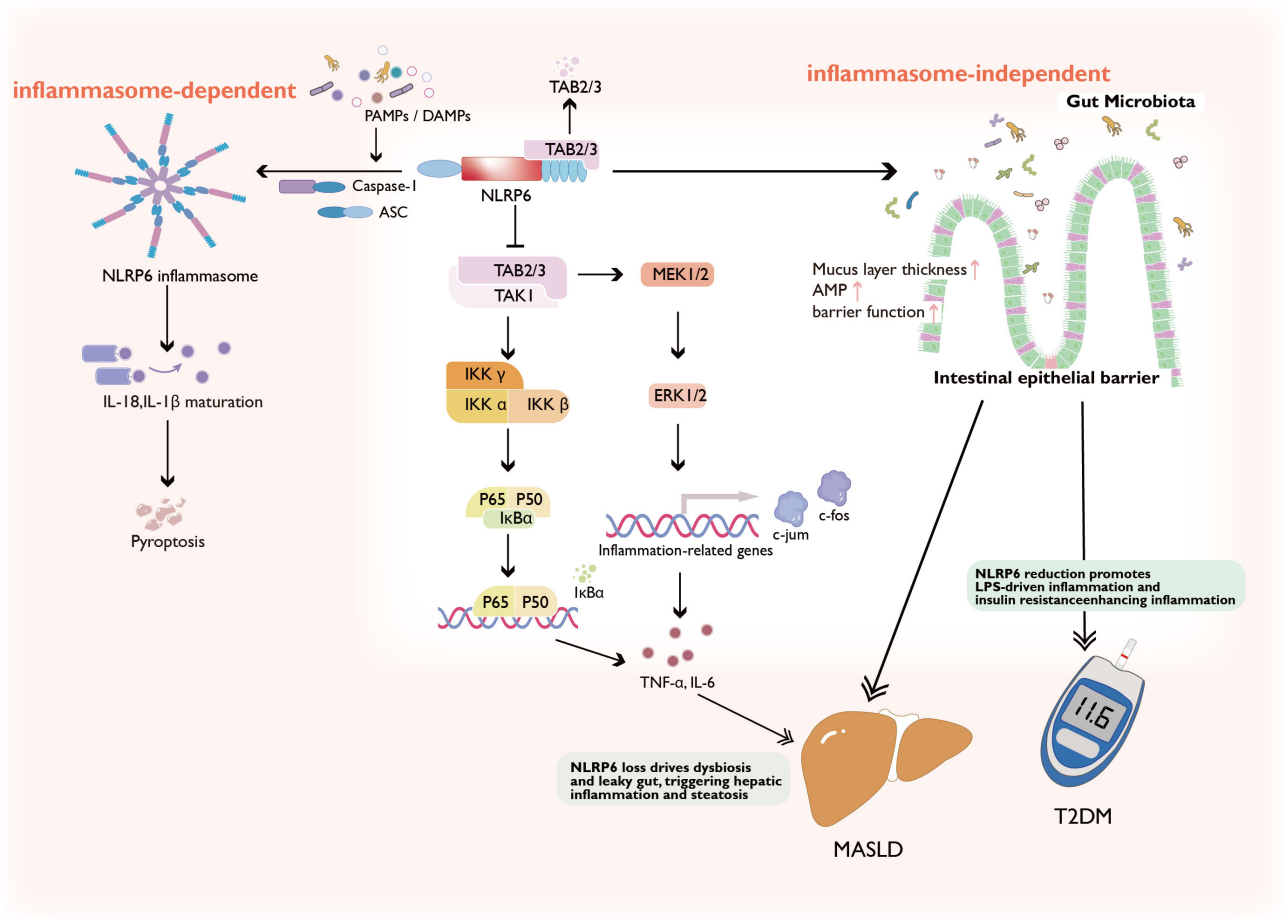
Regulatory roles of NLRP6 in metabolic disorders. NLRP6 regulates intestinal barrier function, inflammatory responses, and gut microbiota homeostasis through inflammasome-dependent and inflammasome-independent pathways. In the inflammasome-dependent pathway, NLRP6 interacts with ASC to promote caspase-1 activation and processing of IL-1β and IL-18, leading to pyroptosis. In inflammasome-independent pathways, NLRP6 modulates NF-κB and MAPK signaling, mucus secretion, antimicrobial peptide production, and gut microbiota composition. Dysregulation of these mechanisms contributes to intestinal dysfunction, MASLD, and T2DM.

NLRP6 regulates inflammatory responses and metabolic disturbances through multiple mechanisms, including both canonical inflammasome activation and non-canonical pathways independent of inflammasome formation. The canonical pathway involves NLRP6 interaction with the adaptor protein ASC, leading to caspase-1 activation and subsequent processing of IL-1β and IL-18. NLRP6 enhances host antiviral defense by eliminating infected cells through pyroptosis, an inflammatory form of programmed cell death that restricts viral replication and spread (69–71). This PAMP/DAMP-responsive system plays vital roles in host defense against microbial pathogens, while its inflammasome-independent functions contribute to infection regulation. The inflammasome-independent pathway also involves NLRP6-mediated inhibition of key signaling cascades, including NF-κB and MAPK pathways. This activity suppresses inflammatory gene expression independently of inflammasome assembly and contributes to intestinal immune equilibrium (72–74) (**Figure 5**).

Furthermore, NLRP6 participates in intestinal antiviral infection in a non-inflammasome-dependent manner: its antiviral function exhibits intestinal specificity and exerts no effect on systemic infections. Specifically, Nlrp6^-^/^-^ mice show increased intestinal viral load, as well as elevated mortality and viremia following oral infection. At the molecular level, NLRP6 binds to viral RNA in association with Dhx15, and subsequently induces the expression of type I/III interferons and IFN-stimulated genes via interaction with MAVS, thereby mediating viral clearance. This function expands the known roles of NLRP6 and identifies it as a key viral RNA sensor in intestinal antiviral innate immunity (75).

#### 3.3.1 NLRP6 in intestinal barrier dysfunction and T2DM

The structural organization of NLRP6 allows it to serve as a versatile regulator at the interface of immunity and metabolism (76). The NLRP6-dependent pathway primarily regulates gut microbiota composition, antimicrobial peptide production, and inflammatory cytokine secretion. Within the intestinal tract, the NLRP6 inflammasome maintains epithelial homeostasis and modulates inflammatory responses. NLRP6-deficient mice exhibit heightened sensitivity to inflammatory stimuli and increased susceptibility to inflammatory bowel disease-like pathology. Mechanistically, studies demonstrate that NLRP6 co-expression with ASC activates caspase-1 and enhances IL-18 secretion, thereby bolstering intestinal immune defense mechanisms (77, 78). Concurrently, NLRP6 maintains gut microbial homeostasis by modulating the composition and thickness of the intestinal mucus layer while stimulating Paneth cells to secrete antimicrobial peptides.

NLRP6 plays multifaceted roles in obesity-associated inflammation. Patients with obesity and T2DM exhibit significantly reduced NLRP6 and IL-18 gene expression in the jejunum, concomitant with increased intestinal permeability and systemic inflammation. Mechanistically, NLRP6 inflammasome activation by commensal microbiota metabolites regulates microbial composition through modulation of mucus secretion and antimicrobial peptide release, thereby maintaining gut homeostasis. Obesity and related metabolic disorders, including T2DM, are characterized by profound alterations in gut microbiota composition and function, evidenced by detectable bacterial components and commensal DNA in circulation and adipose tissue depots. These findings collectively demonstrate that diminished NLRP6 inflammasome expression correlates with both intestinal barrier dysfunction and enhanced inflammation in obese T2DM patients (79). Studies demonstrate that ginsenoside Rk2 (a dehydrated protopanaxadiol saponin) ameliorates alcohol-induced intestinal barrier dysfunction by upregulating colonic NLRP6 expression. This compound further enhances NLRP6 inflammasome activity through modulation of fecal taurine levels, thereby restoring alcohol-suppressed mucus production and antimicrobial peptide secretion. These mechanisms collectively improve intestinal barrier integrity and reduce hepatic translocation of bacterial LPS (80).

#### 3.3.2 NLRP6 in MASLD

In MASLD models, NLRP6 appears to regulate hepatic steatosis and inflammation partly through hepatocyte lipid-metabolism pathways. Hepatocyte-specific deletion of NLRP6 in mice has been reported to exacerbate steatohepatitis-like liver injury and accelerate the progression from simple steatosis to MASH-like inflammatory liver disease, whereas NLRP6 overexpression or preservation attenuates hepatic lipid accumulation and inflammatory signaling. Mechanistically, NLRP6 has been proposed to regulate MASLD/MASH-related pathology by modulating CD36 expression and suppressing NF-κB signaling. In this context, NLRP6 may physically interact with TAB2/3 and promote their degradation, thereby reducing NF-κB phosphorylation and downstream inflammatory activation. Complementary findings from ob/ob mice suggest that NLRP6 overexpression can improve hepatic steatosis and inflammation, supporting a dual role in lipid metabolism and anti-inflammatory regulation (81). In addition, NLRP6 deficiency has been associated with aggravated steatohepatitis and accelerated hepatocellular carcinoma development through gut microbiota dysbiosis, a process that can be attenuated by antibiotic treatment (82). Collectively, these findings support NLRP6 as an emerging, context-dependent regulator of hepatic steatosis and inflammation. However, most causal evidence comes from mouse models or *in vitro* systems, whereas human metabolic-disease data remain largely correlative (79). Therefore, NLRP6 should be presented as a candidate pathway requiring further validation rather than an established therapeutic target for MASLD/MASH.

### 3.4 NLRC5 in antigen presentation and metabolic disease

NLRC5 (NOD-like receptor family CARD domain-containing 5) represents one of the most structurally distinctive members of the NLR family. Its N-terminus features an atypical CARD domain that exhibits significant structural divergence from canonical CARD domains in other NLR proteins. Additionally, NLRC5 contains a central nucleotide-binding domain and an LRR region consisting of 27 repeat units. This represents the longest LRR architecture among all NLR family members, and correspondingly renders NLRC5 the largest protein within this family (83). Although the specific activating ligand of NLRC5 remains unidentified, its biological functions have been extensively characterized. NLRC5 is best recognized as the master transcriptional regulator of MHC I genes. Expressed on all nucleated cells, MHC-I molecules mediate antigen presentation to CD8^+^ T cells, a critical step for cytotoxic T cell activation. Mechanistically, NLRC5 orchestrates MHC I gene expression through cooperative interactions with the regulatory factor X transcription factor complex, thereby playing a central role in adaptive immunity (84, 85). Thus, throughout this review, NLRC5-related metabolic effects are interpreted mainly through transcriptional regulation, antigen presentation, inflammatory signaling, and lipid-metabolic associations, rather than through a canonical inflammasome-sensor framework.

NLRC5 primarily functions within the immune system, where it exhibits pleiotropic regulatory effects in inflammatory and metabolic contexts. Notably, NLRC5 is not recognized as a canonical inflammasome sensor; its best-established biological role is the transcriptional regulation of MHC class I rather than forming a typical inflammasome complex (27, 86). Although a small number of studies have proposed potential inflammasome-related activity of NLRC5, this viewpoint remains controversial and has not been widely validated (28, 87). In limited experimental contexts, NLRC5 may physically interact with NLRP3 and act as a co-activator of the NLRP3 inflammasome, potentiating its inflammatory activity (88).

Upregulation of NLRC5 expression suppresses NF-κB signaling pathway activation, thereby attenuating the secretion of proinflammatory cytokines, including TNF-α, IL-6, and IL-1β, and exerting anti-inflammatory effects (89, 90). NLRC5 has been identified as a negative regulator of type I interferon responses, playing a modulatory role in antiviral immunity (86, 91). Additionally, NLRC5 modulates TLR2-mediated NF-κB signaling, thereby regulating inflammatory responses induced by PAMPs such as lipoteichoic acid (LTA) (90).

**Figure 6:**
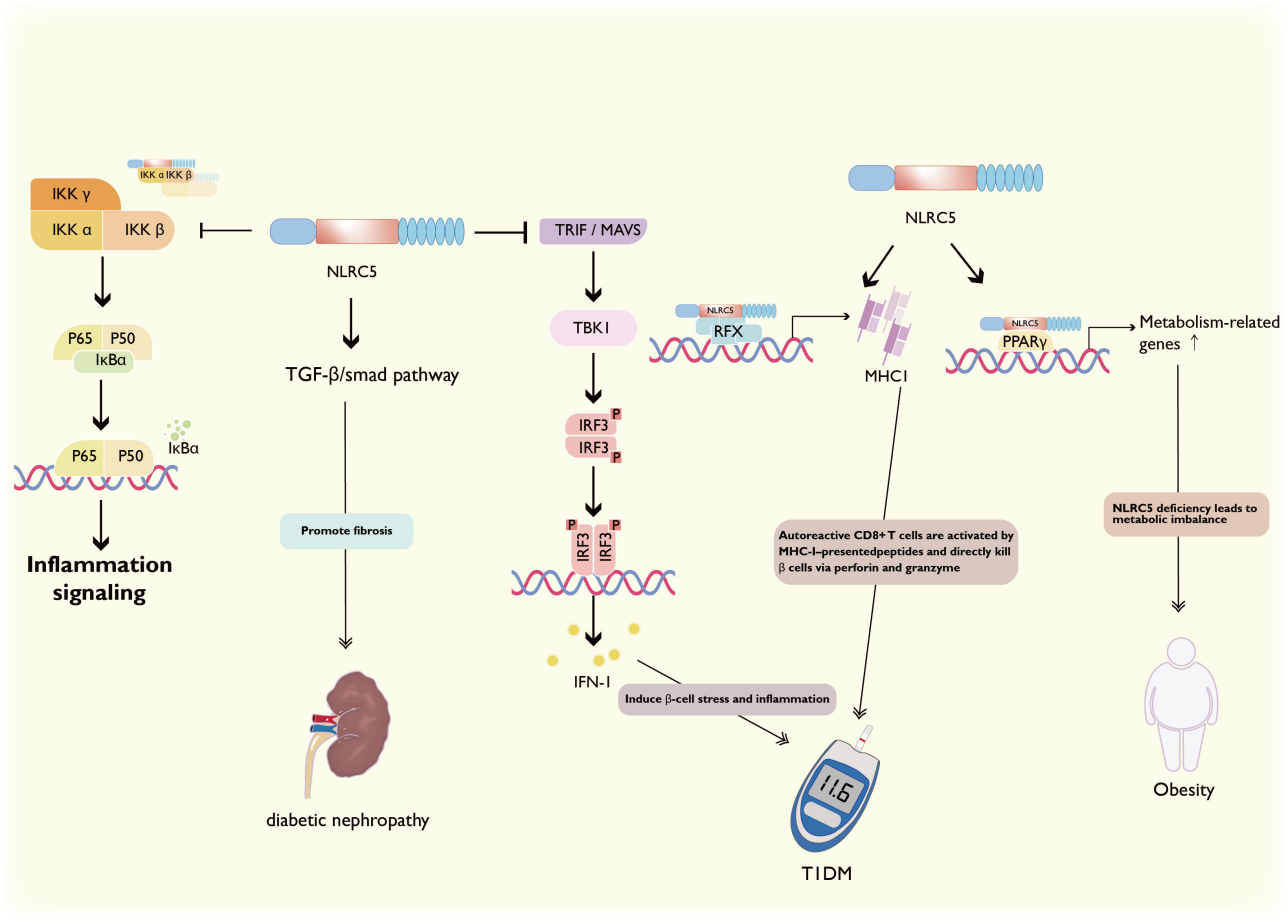
Mechanisms of NLRC5 in metabolic disorders. NLRC5 is a key regulator of MHC class I gene expression and antigen presentation. It also modulates inflammatory signaling pathways, including suppression of NF-κB and negative regulation of type I interferon responses. In metabolic disease contexts, NLRC5 is associated with lipid-metabolic regulation, obesity, diabetes-related antigen presentation, and diabetic nephropathy. NLRC5 cooperates with PPARγ to regulate lipid-metabolic gene expression and influences renal inflammation and fibrosis through TGF-β/Smad signaling.

Emerging evidence has highlighted the involvement of NLRC5 in metabolic disorders, particularly in obesity, lipid metabolism, and insulin sensitivity. Epigenetic studies reveal differential methylation patterns of the NLRC5 gene locus across individuals with varying body weights. Notably, Meeks et al. demonstrated in a Ghanaian population that methylation levels at the NLRC5 locus positively correlate with body mass index (BMI), obesity status, and waist circumference (92). In contrast, Cao et al. observed that obese children exhibit hypomethylation at the NLRC5 locus compared to their normal-weight counterparts (93). These findings suggest NLRC5 may contribute to obesity pathogenesis through epigenetic mechanisms. Furthermore, NLRC5 has been identified as a candidate gene influencing high-density lipoprotein (HDL) levels in humans (94). Single-nucleotide polymorphisms (SNPs) within the NLRC5 promoter region show significant associations with elevated triglyceride levels and dyslipidemia, suggesting its regulatory role in lipid metabolism (95). Notably, NLRC5 has been shown to functionally cooperate with peroxisome proliferator-activated receptor γ (PPARγ) to enhance expression of lipid metabolic target genes, thereby orchestrating adipogenic and lipogenic processes (96) (**Figure 6**).

NLRC5 serves as the master transcriptional regulator of IFN-α-mediated antigen presentation in pancreatic β-cells, potentially facilitating their immune recognition and destruction during early T1DM pathogenesis. Experimental studies demonstrate that NLRC5 knockout substantially reduces IFN-α-induced expression of HLA-ABC and related antigen presentation genes in human pancreatic β-cells (97). Similarly, studies reveal that NLRC5 deficiency ameliorates diabetic nephropathy by attenuating inflammatory responses, potentially through modulation of both NF-κB and TGF-β/Smad signaling pathways, thereby suppressing renal inflammation and fibrosis (98).

While the role of NLRC5 in immune regulation and cell death has been extensively characterized, its precise mechanisms in obesity and metabolic diseases remain incompletely understood. Current evidence suggests that NLRC5 may be associated with obesity, diabetes, and related complications through regulation of antigen presentation, inflammatory signaling, and lipid-metabolic pathways. However, causal evidence in human metabolic disease is limited, and several observations remain epigenetic or association-based (92–95, 97). Future investigations should clarify the tissue-specific and disease-context-dependent functions of NLRC5 and evaluate whether these associations can be translated into therapeutic strategies for obesity-associated metabolic disorders.

### 3.5 NLRP12 in inflammation and metabolic disease

NLRP12, also known as NALP12, Monarch-1, PYPAF7, or RNO, is a significant member of the NLR family, predominantly expressed in myeloid monocytes. NLRP12 functions as a negative regulator of inflammatory responses via multiple mechanisms to inhibit both conventional and noncanonical NF-κB signaling pathways, in addition to suppressing extracellular signal-regulated kinase (ERK) activation, thus playing a crucial role in the regulation of inflammation (99, 100). NLRP12 directly interacts with IRAK1, an essential kinase in the conventional NF-κB pathway, and TRAF3, a pivotal adaptor protein in the noncanonical NF-κB pathway, to limit NF-κB activation and hence attenuate inflammatory responses (101, 102). Mutations in NLRP12 impair its ability to suppress NF-κB signaling, leading to unregulated inflammatory responses, notably shown in NLRP12-associated autoinflammatory disease (NLRP12-AID) (103).

**Figure 7:**
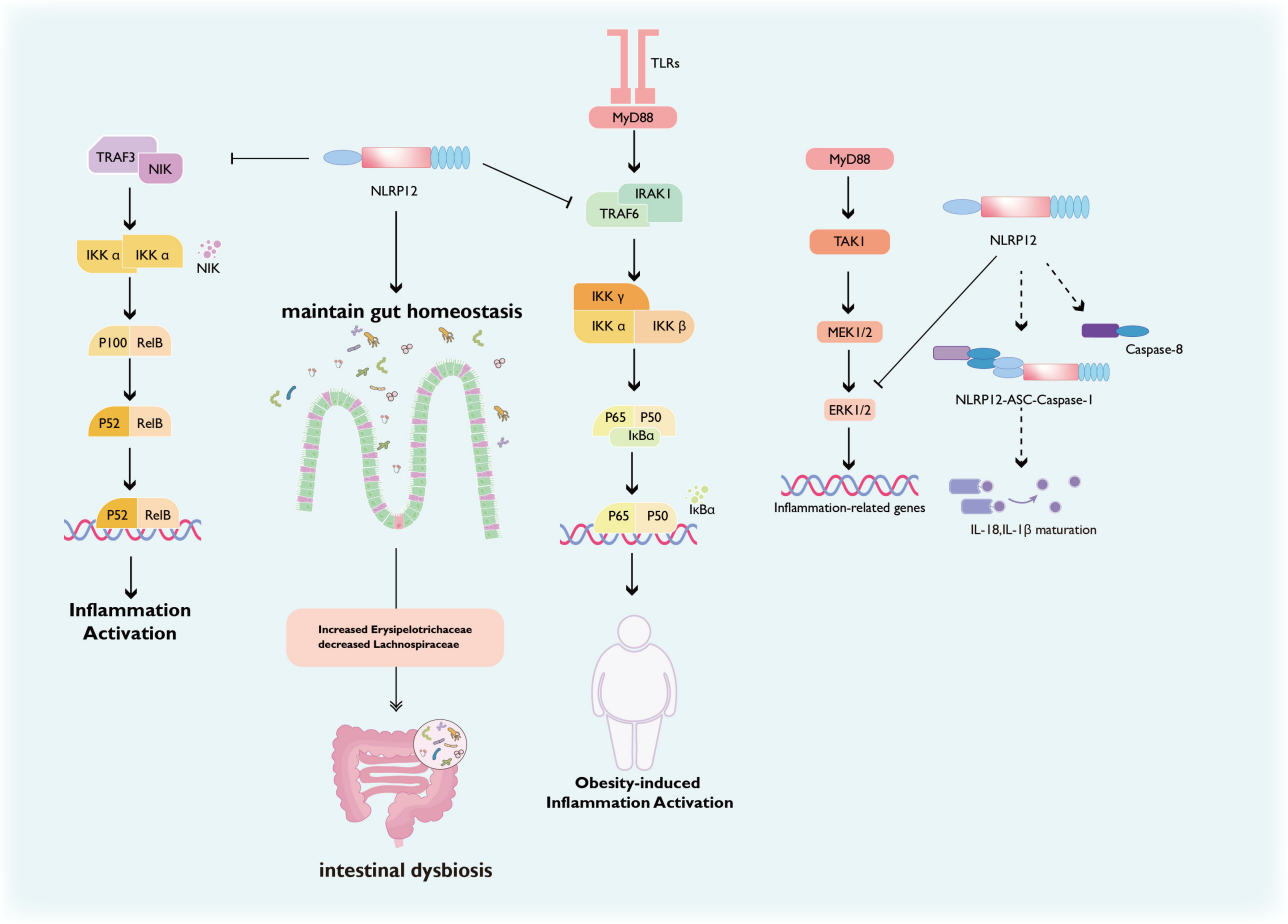
Regulatory roles of NLRP12 in inflammation and metabolic disease. NLRP12 negatively regulates inflammatory responses by inhibiting both canonical and noncanonical NF-κB signaling, as well as the ERK pathway, via interactions with IRAK1 and TRAF3. It also participates in inflammasome assembly, promoting caspase-1 activation and maturation of IL-1β and IL-18, with potential crosstalk to caspase-8. NLRP12 maintains gut microbiota homeostasis, limiting intestinal dysbiosis and obesity-induced inflammation.

In addition to its well-described role as a negative regulator of NF-κB and ERK signaling, NLRP12 has been reported in selected experimental settings to participate in inflammasome assembly and PANoptosis-related cell-death complexes (**Figure 7**). In these contexts, NLRP12 may contribute to caspase-1 activation and the maturation of IL-1β and IL-18, and may also interact with cell-death effectors such as caspase-8 and RIPK3 to coordinate crosstalk among pyroptosis, apoptosis, and necroptosis (104). However, these functions appear to be highly context dependent, and whether NLRP12-driven inflammasome or PANoptosis-related mechanisms operate broadly in metabolic disease remains uncertain. Therefore, NLRP12 should be discussed as a context-dependent regulator with both anti-inflammatory and pro-cell-death activities, rather than as a uniformly proinflammatory PANoptosome sensor.

In metabolic disease models, emerging evidence supports a protective role of NLRP12 in maintaining gut microbiota homeostasis and limiting diet-induced obesity. Gut microbiota imbalance (dysbiosis) is closely associated with intestinal inflammation. NLRP12-deficient mice developed more severe obesity, inflammation, and insulin resistance under high-fat diet conditions, which was strongly associated with gut dysbiosis (characterized by increased *Erysipelotrichaceae* and decreased *Lachnospiraceae*). Supplementation with *Lachnospiraceae* or their metabolic product short-chain fatty acids (SCFAs) effectively reversed the metabolic abnormalities caused by NLRP12 deficiency. A high-fat diet alters bile acid composition, further compromising intestinal barrier function. Notably, metabolites from obesity-associated bacterial strains such as *Erysipelotrichaceae* directly suppress anti-inflammatory NLRs, including NLRP12, or activate NLRP3. Conversely, SCFAs like butyrate and propionate exhibit potent anti-inflammatory effects by inhibiting HDAC activity, activating GPCRs such as GPR43, and maintaining gut barrier integrity, thereby indirectly suppressing NLRP3 activation. Clinical studies demonstrate that butyrate supplementation improves insulin sensitivity in obese patients (105). Fecal microbiota transplantation from NLRP12-deficient mice to germ-free mice successfully transferred the obesity and inflammatory phenotypes (106). NLRP12 helps maintain intestinal microbial homeostasis by regulating gut microbiota composition, thereby contributing to obesity prevention (107).

Collectively, current evidence suggests that NLRP12 exerts context-dependent effects in inflammation and metabolic disease. In mouse models of diet-induced obesity, NLRP12 appears to play a protective role by preserving gut microbial balance and limiting chronic low-grade inflammation (107). However, human evidence for NLRP12-related metabolic disease remains limited and is mainly association-based rather than causal (99, 108). Evidence from NLRP12-associated autoinflammatory disease supports the clinical relevance of NLRP12 dysregulation but does not directly establish its causal role in metabolic disease (103).

### 3.6 NLRP2 in MASLD-related metabolic inflammation

NLRP2 (NOD-like receptor family pyrin domain-containing 2), a member of the NLR family, has been implicated in the regulation of inflammatory responses, metabolic homeostasis, and cellular differentiation. Current evidence suggests that NLRP2 may exert regulatory functions across several physiological and pathological processes, including MASLD-related metabolic inflammation, metabolic syndrome, immune regulation, and cellular differentiation. NLRP2 overexpression has been reported to suppress NF-κB signaling activation and negatively regulate type I interferon responses, suggesting a possible negative-feedback mechanism that limits excessive inflammatory activation (109). Mechanistically, NLRP2 may suppress activation of the NF-κB p65 signaling pathway through interactions with proteins involved in IκB regulation (110). Under TNF-α stimulation, NLRP2 deficiency enhances NF-κB p65 activation, further supporting its potential role in inflammatory regulation.

**Figure 8:**
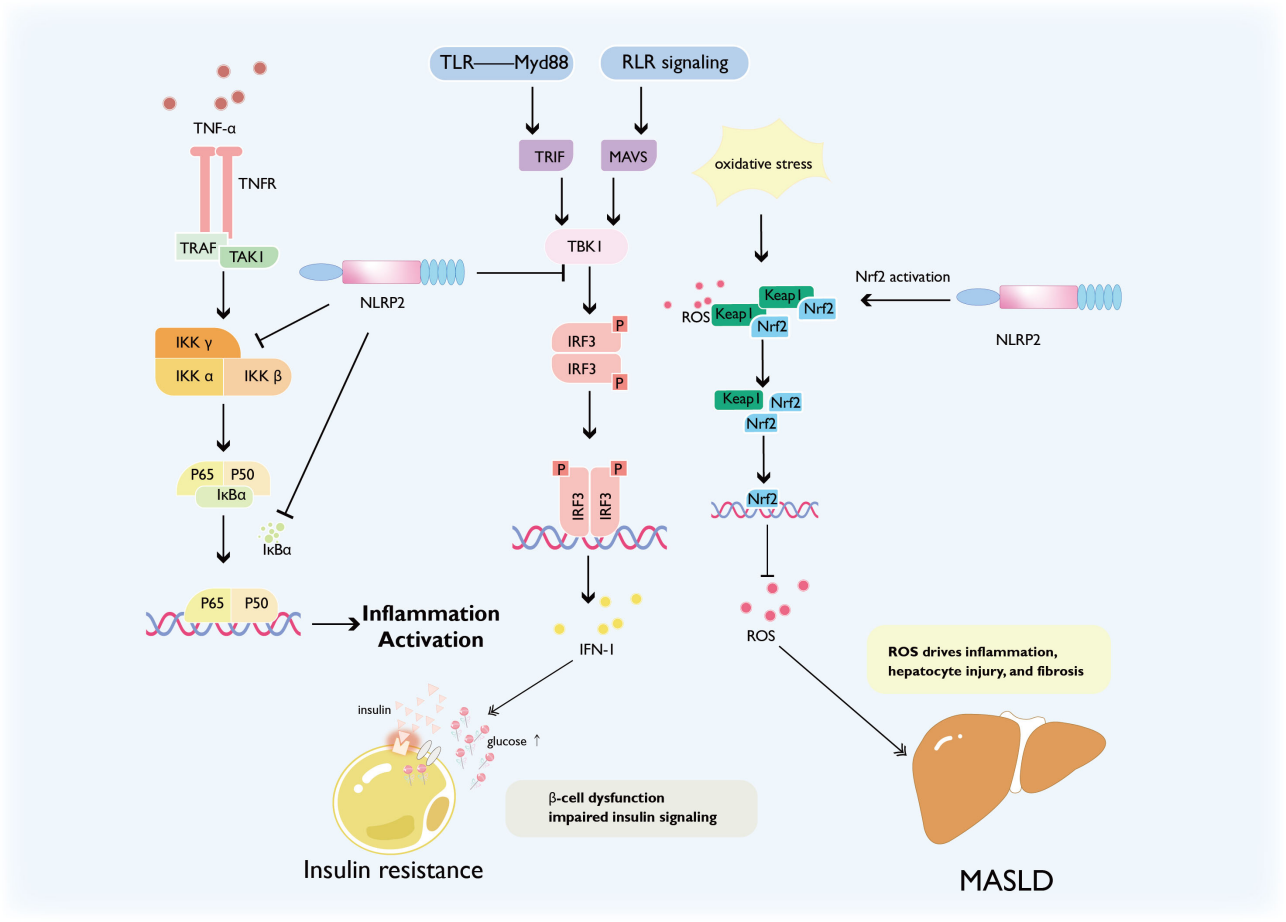
Regulatory pathways of NLRP2 in metabolic inflammation.NLRP2 regulates inflammatory signaling, metabolic homeostasis, and oxidative stress responses. NLRP2 suppresses NF-κB signaling and type I interferon responses to limit excessive inflammatory activation. In MASLD-related metabolic inflammation, NLRP2 activates Nrf2-mediated antioxidant signaling, mitigating hepatic inflammation, oxidative stress, and metabolic dysfunction.

The role of NLRP2 in metabolic disease has received increasing attention. Reduced NLRP2 expression has been reported in liver tissues from patients with MASLD, and in HFD-induced MASLD mouse models, NLRP2 deficiency exacerbates hepatic inflammation and oxidative stress, thereby aggravating metabolic dysfunction and insulin resistance (111). Mechanistic studies suggest that NLRP2 deficiency may enhance oxidative stress and inflammatory responses partly through suppression of the Nrf2 signaling pathway (**Figure 8**). Activation of Nrf2, a master regulator of antioxidant responses, can mitigate NLRP2 deficiency-associated inflammation and ROS production (111). Consequently, NLRP2 may represent a potential protective regulator in experimental MASLD models. However, current evidence is still limited, with mechanistic support mainly from cell and mouse studies and only limited human validation. Its therapeutic relevance therefore remains exploratory (109, 112).

Recent findings also suggest that NLRP2 may be involved in inflammatory diseases such as periodontitis and diabetes through modulation of inflammatory signaling pathways (113). Nevertheless, the molecular mechanisms of NLRP2 remain incompletely understood, and its tissue-specific functions across different organs require further clarification. Future studies should define the context-dependent roles of NLRP2 in innate immunity, inflammatory diseases, and metabolic disorders before its therapeutic potential can be reliably evaluated.

Taken together, the selected NLR family members discussed above differ substantially in disease context, cellular localization, inflammasome dependence, direction of disease modulation, and strength of supporting evidence. We summarize and compare these NLRs by highlighting their metabolic disease associations, primary inflammasome-dependent or inflammasome-independent functions, subcellular localizations, functional roles in disease progression, levels of evidence, and therapeutic implications (**Table 1**).

**Table 1:** Integrative summary of selected NLR family members in metabolic diseases.

NLR member	Metabolic disease context	Predominant inflammasome-dependent vs inflammasome-independent functions	Main cellular compartment	Disease-promoting vs disease-limiting role	Evidence level	Therapeutic implications
NLRP3	Obesity, T2DM, MASLD/MASH, atherosclerosis, diabetic complications	Mainly inflammasome-dependent; caspase-1–IL-1β/IL-18–GSDMD axis; limited inflammasome-independent effects reported	Macrophages, adipocytes, hepatocytes, HSCs, endothelial cells, VSMCs, β-cells	Mostly disease-promoting; inhibition may be disease-limiting	Strong	Most advanced target; NLRP3 inhibitors and IL-1β/IL-1R blockade are promising, but safety and tissue specificity require evaluation. Refs (7, 29–60, 114–138):
NLRP1	Obesity, metabolic syndrome, diabetes-related immune dysregulation	Inflammasome-dependent IL-18 processing; regulated by CARD8/DPP9 axis	Adipose tissue, liver, skeletal muscle, immune cells, T cells/islet microenvironment	Context-dependent; IL-18 may limit obesity, whereas dysregulation may promote inflammation	Moderate to limited	Potential immunometabolic regulator; therapeutic targeting remains preliminary. Refs (61–68):
NLRP6	Obesity-associated inflammation, T2DM-related gut barrier dysfunction, MASLD/MASH	Inflammasome-dependent and independent; regulates IL-18, mucus/AMP secretion, gut microbiota, NF-κB/MAPK signaling	Intestinal epithelial cells, goblet cells, Paneth cells, hepatocytes, gut–liver axis	Often disease-limiting through gut barrier, microbiota, and hepatic anti-inflammatory effects	Moderate	Candidate gut–liver/metabolism pathway; not established as a MASLD/MASH therapeutic target. Refs (69–82):
NLRC5	Obesity, lipid metabolism, insulin sensitivity, diabetes-related antigen presentation, diabetic nephropathy	Mainly inflammasome-independent; MHC-I transcriptional regulator; possible NLRP3/PANoptosis-related effects remain limited	Nucleated cells, immune cells, β-cells, renal cells, adipose/metabolic tissues	Context-dependent; metabolic role mostly association-based	Emerging/association-based	Not a canonical inflammasome target; therapeutic relevance should be interpreted through antigen presentation, transcriptional regulation, and lipid-metabolic associations. Refs (27, 28, 83–98):
NLRP12	Obesity, gut dysbiosis, insulin resistance, autoinflammatory contexts	Inflammasome-independent NF-κB/ERK inhibition; selected models suggest inflammasome/PANoptosis-related activity	Myeloid cells, monocytes/macrophages, intestinal immune compartments	Context-dependent; protective in diet-induced obesity, but may participate in cell-death complexes in selected settings	Moderate in mouse models; limited in human metabolic disease	Candidate immunometabolic regulator; not a uniformly proinflammatory PANoptosome sensor or established metabolic therapeutic target. Refs (25, 99–108):
NLRP2	MASLD/MASH-related inflammation, metabolic syndrome, diabetes/periodontitis-associated inflammation	Mainly inflammasome-independent; may regulate NF-κB, IFN-I, and Nrf2-related oxidative stress	Hepatocytes/liver tissue, immune cells, inflammatory tissues	Potentially protective in experimental MASLD models	Limited/emerging	Exploratory protective regulator; therapeutic relevance requires human validation. Refs (109–113):

Evidence level was categorized qualitatively according to the overall strength of available data discussed in this review. Strong indicates support from human tissue or intervention data together with animal and mechanistic studies; moderate indicates mainly animal and mechanistic evidence with limited human association data; limited/emerging indicates evidence mainly from cell or mouse studies, or human association/epigenetic data without direct causal validation.

## 4 Therapeutic strategies targeting NLR inflammasome pathways

Based on the evidence hierarchy summarized above, therapeutic strategies targeting NLR inflammasome pathways can be divided into four major categories: direct inflammasome/NLR inhibitors, downstream cytokine blockade, indirect modulators of inflammasome activation, and natural products or multi-target agents. Because the strength of evidence differs substantially across these categories, this section distinguishes clinically validated anti-inflammatory therapies from investigational small molecules, preclinical compounds, and natural products supported mainly by cell or animal studies. Representative therapeutic strategies according to therapeutic category, mechanism of action, evidence level, clinical status, and major translational limitations are summarized (**Table 2****).**

**Table 2:** Evidence-graded summary of representative therapeutic strategies targeting NLR inflammasome pathways in metabolic disease.

Category	Representative agents/strategies	Main target/mechanism	Evidence/clinical status	Major limitations/translational considerations	Refs.
Direct inflammasome/NLR inhibitors	MCC950 and derivatives	Inhibit NLRP3 ATPase activity or improve MCC950-like pharmacokinetic/safety profiles	Strong preclinical; limited clinical translation	Benchmark NLRP3 inhibitors, but clinical translation is limited by hepatotoxicity and lack of metabolic-disease validation	(114–116)
	CY-09	Blocks the ATP-binding site of NLRP3 and suppresses NLRP3 ATPase activity	Preclinical metabolic-disease models	Human safety and metabolic efficacy remain unestablished	(117, 118)
	Tranilast	Binds the NLRP3 NACHT domain and interferes with NLRP3 oligomerization	Repurposing evidence; limited clinical data in diabetic nephropathy	Low oral bioavailability and gastrointestinal adverse effects limit translation	(119, 120)
	Oridonin / INF39	Covalent NLRP3 inhibition; disruption of NLRP3–NEK7 interaction or NLRP3 activation	Preclinical	Covalent mechanisms raise off-target concerns; human metabolic-disease data are lacking	(121–123, 130, 131)
	Dapansutrile (OLT1177)	Oral NLRP3 inhibitor suppressing NLRP3 ATPase activity and ASC oligomerization	Early clinical; Dapan-Dia relevant to T2DM/inflammation	Long-term metabolic efficacy and safety require adequately powered clinical trials	(124–126)
	Entrectinib / VX-765	Blocks NEK7–NLRP3 interaction or inhibits caspase-1-mediated IL-1β/IL-18 maturation	Preclinical for metabolic disease; prior non-metabolic clinical experience for VX-765	Metabolic benefits remain animal-model based; safety concerns such as liver enzyme elevation require caution	(127–129)
Downstream cytokine blockade	Anakinra	IL-1 receptor antagonist blocking IL-1α/IL-1β signaling	Clinical trial evidence in T2DM-related settings	Injection-site reactions and infection risk; not broadly established as metabolic therapy	(132–134)
	Canakinumab	Monoclonal antibody neutralizing IL-1β	Large randomized cardiovascular evidence; limited diabetes-prevention efficacy	Did not prevent diabetes progression; fatal infection risk limits broad chronic metabolic use	(135–137)
	Gevokizumab / anti-TNF-α therapy	IL-1β or TNF-α blockade	Early clinical or inconsistent observational evidence	Evidence is weaker than CANTOS-scale trials; infection/reactivation risks and disease-context dependence remain concerns	(138, 139)
Indirect modulators	Colchicine	Modulates microtubules, P2X7-related signaling, K^+^ efflux, and inflammasome positioning	Small metabolic studies plus stronger cardiovascular evidence	Cardiovascular approval should not be interpreted as approval for obesity, insulin resistance, or T2DM treatment	(140–144)
	Taurine / BI-1	Regulate taurine flux, ER stress, and NLRP3-associated pyroptosis or β-cell stress responses	Preclinical; indirect human metabolic evidence for taurine	Causal links between inflammasome inhibition and human metabolic improvement remain indirect	(145–148)
	Arachidonic acid / PPA-related strategies	Modulate PLC/JNK1-related NLRP3 activation or NLRP3 palmitoylation pathways	Preclinical or conceptual	Biological effects are context-dependent; PPA-targeting strategies remain theoretical	(149–151)
Natural products or multi-target agents	Berberine / Resveratrol	Suppress NLRP3-related inflammation, macrophage pyroptosis, or metabolic stress pathways	Preclinical plus meta-analysis evidence	Clinical evidence is heterogeneous; formulation, dose, follow-up, and safety reporting vary	(152–159)
	Ginsenoside Rb2 / Salidroside	Modulate NLRP3-associated pyroptosis, P2X7/NLRP3, or AMPK–NLRP3 pathways	Preclinical; limited human evidence outside metabolic-disease endpoints	Poor bioavailability or lack of metabolic-disease-specific trials limits translation	(160–164)
	Brazilin, Genipin, HYSJD, Artesunate, Angoroside C, Calenduloside E, BMA	Multi-target modulation of NLRP3, NF-κB, STAT3, AMPK, SIRT2, oxidative stress, or pyroptosis-related pathways	Mainly preclinical or multi-omics evidence	Metabolic therapeutic relevance remains unvalidated; bioavailability, formulation standardization, reproducibility, and long-term safety require validation	(165–174)

### 4.1 Direct inflammasome/NLR inhibitors

Direct inflammasome inhibitors suppress inflammasome assembly and IL-1β/IL-18 release by targeting NLRP3 ATPase activity, the NACHT domain, NLRP3–NEK7 interaction, or downstream caspase-1 activity. In preclinical metabolic disease models, these compounds have been reported to improve glucose metabolism, insulin resistance, hepatic steatosis, vascular inflammation, or renal fibrosis. However, most direct NLRP3 inhibitors remain at the preclinical or early clinical stage. MCC950 is widely used as a benchmark NLRP3 inhibitor but its clinical development was limited by hepatotoxicity, whereas covalent inhibitors such as oridonin and INF39 require careful evaluation because of potential off-target effects. Among direct NLRP3 inhibitors, dapansutrile (OLT1177) is one of the most clinically advanced agents and has been evaluated in clinical studies, including metabolic or inflammation-related indications.

#### 4.1.1 MCC950

MCC950 is a selective small-molecule NLRP3 inflammasome inhibitor. Mechanistically, it binds to the Walker B motif of the NLRP3 NACHT domain and inhibits ATP hydrolysis, thereby stabilizing NLRP3 in an inactive conformation and preventing inflammasome assembly. MCC950 inhibits both canonical and non-canonical NLRP3 activation at nanomolar concentrations without directly inhibiting AIM2, NLRC4, or NLRP1 inflammasomes (114).

*In vitro*, MCC950 blocks NLRP3-dependent caspase-1 activation and IL-1β secretion in LPS-primed mouse bone marrow-derived macrophages and human monocytes. In preclinical studies, MCC950 has been evaluated in multiple disease models, including high-fat diet-induced metabolic dysfunction, db/db diabetic mice, streptozotocin-induced diabetic nephropathy, atherosclerosis, liver injury, and neuroinflammatory disease models. These studies suggest potential benefits in reducing inflammatory cytokine production, improving metabolic parameters, attenuating hepatic steatosis, and reducing renal inflammation or fibrosis (115).

However, clinical translation of MCC950 has been limited by dose-dependent hepatotoxicity observed during early clinical development. This toxicity has been linked to reactive metabolite formation involving structural moieties such as the furan ring and sulfonylurea group. Therefore, MCC950 is best considered a benchmark compound for mechanistic and preclinical validation rather than a clinically ready therapy for metabolic disease. Structural derivatives such as DFV890 and N14 have been developed to improve pharmacokinetic and hepatic safety profiles while retaining NLRP3 inhibitory potency, but their efficacy and safety in metabolic disease still require clinical validation (116).

#### 4.1.2 CY-09

CY-09 is a direct NLRP3 inhibitor that competitively occupies the ATP-binding site of the Walker A motif, thereby suppressing NLRP3 ATPase activity and blocking inflammasome assembly (117). *In vitro*, CY-09 specifically inhibits NLRP3 inflammasome activation in LPS-primed macrophages without affecting AIM2 or NLRC4 inflammasomes.

In animal studies, treatment of high-fat diet-induced diabetic mice and cryopyrin-associated periodic syndrome models with CY-09 reduced blood glucose and insulin levels, improved glucose tolerance, attenuated hepatic steatosis, and decreased IL-1β levels in serum, adipose tissue, and liver (118). CY-09 has shown favorable oral bioavailability and no obvious cardiotoxicity in preclinical models, although mild inhibition of selected cytochrome P450 enzymes has been reported. Human safety and metabolic efficacy remain to be established.

#### 4.1.3 Tranilast

Tranilast is a clinically used anti-allergic drug that has been reported to inhibit NLRP3 inflammasome activation by directly binding to the NLRP3 NACHT domain and interfering with NLRP3 oligomerization (119). *In vitro*, tranilast concentration-dependently inhibits NLRP3 activation in primary macrophages, with little effect on AIM2 or NLRC4 inflammasomes.

In animal models, tranilast reduced body weight and fasting blood glucose in high-fat diet-fed diabetic mice, and these effects were abolished in Nlrp3-deficient mice, supporting an NLRP3-dependent mechanism. Tranilast has also shown activity in gouty arthritis and CAPS models and has demonstrated ex vivo activity in synovial fluid monocytes from patients with gout. Clinically, tranilast has been investigated in diabetic nephropathy, and pooled analyses suggest that tranilast may slow the progression of advanced diabetic nephropathy (120). Its long clinical use as an anti-allergic agent provides some safety experience, but its relatively low oral bioavailability and gastrointestinal adverse effects at higher doses remain important translational limitations.

#### 4.1.4 Oridonin

Oridonin is a natural diterpenoid compound isolated from Rabdosia/Isodon species. Mechanistically, oridonin covalently modifies Cys279 in the NLRP3 NACHT domain and disrupts the NLRP3–NEK7 interaction, thereby inhibiting NLRP3 inflammasome assembly (121).

*In vitro*, oridonin inhibits NLRP3 activation in high-glucose-stimulated human retinal endothelial cells, improves cell viability, and reduces IL-1β release, partly through the suppression of the NEK7–NLRP3 interaction. In animal studies, oridonin reduced body weight and blood glucose in high-fat diet-fed diabetic mice, and these effects were absent in Nlrp3-deficient mice. In streptozotocin-induced diabetic retinopathy models, oridonin alleviated visual dysfunction, reduced retinal and vascular lesions, protected neuroretinal structure, and suppressed the NEK7–NLRP3 interaction (122, 123).

Because oridonin acts through covalent modification, potential off-target effects require careful evaluation. In addition, human pharmacokinetic and safety data in metabolic disease remain insufficient, and its clinical translation should therefore be interpreted cautiously.

#### 4.1.5 Dapansutrile (OLT1177)

Dapansutrile, also known as OLT1177, is an orally active β-sulfonyl nitrile compound that directly inhibits NLRP3 by suppressing NLRP3 ATPase activity and ASC oligomerization, without directly inhibiting AIM2 or NLRC4 inflammasomes (124).

*In vitro*, dapansutrile suppresses NLRP3-dependent IL-1β release in human monocytes and macrophages in a concentration-dependent manner. In animal models, it has shown anti-inflammatory activity and may reduce the metabolic burden associated with inflammation. Clinically, dapansutrile has completed Phase I studies with a favorable safety profile and no reported hepatotoxicity. It has subsequently been evaluated in inflammatory conditions such as acute gout flares and heart failure (125, 126).

Of particular relevance to metabolic disease, the Dapan-Dia study is evaluating dapansutrile in patients with type 2 diabetes mellitus and systemic inflammation or diabetes-related complications. Compared with covalent or irreversible inhibitors, dapansutrile’s reversible and non-covalent pharmacological profile may offer potential safety advantages, although its long-term metabolic efficacy still requires confirmation in adequately powered clinical trials.

#### 4.1.6 Entrectinib

Entrectinib is a multi-target receptor tyrosine kinase inhibitor approved for TRK-, ROS1-, and ALK-fusion-positive cancers. Recent experimental evidence indicates that entrectinib can directly bind Arg121 of NEK7 and sterically block the NEK7–NLRP3 interaction, thereby inhibiting NLRP3 inflammasome activation without directly affecting NLRC4 or AIM2 inflammasomes (127).

*In vitro*, entrectinib selectively suppresses NLRP3 inflammasome activation. In animal models, it protects against LPS-induced systemic inflammation and MSU-induced peritonitis and has also been reported to ameliorate metabolic inflammation and improve glucose tolerance in high-fat diet-induced T2DM mice. Because entrectinib is already approved for oncological indications, clinical safety information is available, including adverse events such as dysgeusia, dizziness, fatigue, weight gain, and hyperuricemia. However, its metabolic benefits have so far been demonstrated mainly in animal models and have not been clinically validated in patients with metabolic disease.

#### 4.1.7 VX-765

VX-765, also known as belnacasan, is a selective caspase-1 inhibitor that blocks the maturation of IL-1β and IL-18 downstream of inflammasome activation. It may also indirectly suppress NLRP3 activity by promoting Parkin-dependent mitophagy and efferocytosis (128).

*In vitro*, VX-765 inhibits caspase-1-mediated IL-1β production and GSDMD cleavage in macrophages. In animal models of atherosclerosis, VX-765 suppresses plaque progression and vascular inflammation in ApoE-deficient and Ldlr-deficient mice without substantially affecting plasma lipoprotein levels. In a T2DM rat model of cardiopulmonary bypass-induced myocardial injury, VX-765 attenuated myocardial damage and inflammatory cascade activation (129). However, in Phase II clinical studies for epilepsy, long-term administration was associated with elevated liver enzymes, and clinical evidence in metabolic disease remains lacking. Therefore, its translational relevance in metabolic disease remains preclinical.

#### 4.1.8 INF39

INF39 is an orally active acrylate-based covalent NLRP3 inhibitor that suppresses inflammasome activation through irreversible covalent interaction with NLRP3 and partial inhibition of LPS-induced pro-inflammatory gene expression (130).

*In vitro*, INF39 inhibits caspase-1 activation and IL-1β release in LPS-primed BMDMs stimulated with ATP or nigericin. In animal studies, INF39 has shown anti-inflammatory effects in models such as DNBS-induced colitis and has been reported to reduce platelet activation, endothelial adhesion, and vascular inflammation in a T2DM rat model (131). Although covalent binding may prolong pharmacological activity, it also increases the potential risk of off-target effects. Because clinical data are not yet available, the translational relevance of INF39 in metabolic disease remains uncertain.

### 4.2 Downstream cytokine blockade

Downstream cytokine blockade targets inflammatory mediators released after inflammasome activation rather than the inflammasome complex itself. Representative approaches include IL-1 receptor antagonism, IL-1β neutralization, and TNF-α blockade. Compared with direct inflammasome inhibitors, cytokine-targeted therapies have stronger clinical evidence in selected inflammatory or cardiovascular contexts. However, the metabolic benefits are variable, and immune suppression-related risks, especially infection risk, must be considered.

#### 4.2.1 Anakinra

Anakinra is a recombinant human IL-1 receptor antagonist that competitively blocks IL-1R1 and inhibits both IL-1α and IL-1β signaling. It has been approved for inflammatory diseases such as rheumatoid arthritis. In a randomized, double-blind, placebo-controlled trial in patients with T2DM, 13 weeks of anakinra treatment reduced HbA1c by 0.46% and improved C-peptide secretion and the proinsulin-to-insulin ratio, suggesting improved β-cell function (132).

Subsequent studies further suggested that anakinra may improve first-phase insulin secretion in individuals with impaired glucose tolerance (133). The TRACK study showed metabolic and inflammatory benefits of anakinra in patients with rheumatoid arthritis and comorbid T2DM (134). Common adverse effects include injection-site reactions, and a modest increase in infection risk should be considered during long-term use.

#### 4.2.2 Canakinumab

Canakinumab is a selective humanized monoclonal antibody targeting IL-1β and has been approved for several autoinflammatory syndromes, including CAPS. In the CANTOS trial, involving 10,061 patients with prior myocardial infarction and elevated hsCRP, canakinumab significantly reduced major adverse cardiovascular events, providing large-scale randomized evidence that anti-inflammatory therapy can reduce cardiovascular events independently of lipid lowering (135).

However, a prespecified diabetes analysis of CANTOS showed that canakinumab did not prevent progression from prediabetes to overt diabetes, suggesting that IL-1β blockade alone may be insufficient to halt diabetes development (136). In the main CANTOS trial, canakinumab reduced recurrent cardiovascular events but was associated with a higher incidence of fatal infection than placebo (137). Therefore, although canakinumab provides strong proof-of-concept for inflammatory risk reduction, its role in metabolic disease management remains limited by safety considerations and disease-specific efficacy.

#### 4.2.3 Gevokizumab

Gevokizumab is an anti-IL-1β monoclonal antibody. In a Phase II clinical trial in patients with T2DM, gevokizumab improved glycemic control and inflammatory parameters, including reductions in HbA1c and systemic inflammatory markers, with an overall favorable safety profile (138). However, the available evidence remains limited compared with large cardiovascular outcome trials, and further studies are needed to define its long-term efficacy and safety in metabolic disease.

#### 4.2.4 Anti-TNF-α therapy

TNF-α is a key inflammatory mediator implicated in obesity-associated insulin resistance. Anti-TNF-α monoclonal antibodies are widely used in autoimmune diseases such as rheumatoid arthritis and psoriatic arthritis. Case reports and animal studies have suggested possible improvements in glucose metabolism with TNF-α blockade, but clinical evidence in metabolic disease remains inconsistent.

A large cohort study of patients with rheumatoid arthritis reported that initiation of infliximab or adalimumab was associated with a higher risk of diabetes compared with abatacept, suggesting that the metabolic effects of TNF-α blockade may depend on disease context, drug class, and immune-metabolic status (139). Major adverse effects include serious infections, reactivation of latent tuberculosis, infusion reactions, and rare lymphoma risk. Therefore, anti-TNF-α therapy is not recommended as a routine metabolic anti-inflammatory strategy in patients without comorbid autoimmune disease.

### 4.3 Indirect regulators of inflammasome activation

Indirect regulators modulate upstream signals or intracellular conditions required for inflammasome activation rather than directly binding NLRP3 itself. These mechanisms include regulation of K^+^ efflux, mitochondrial dysfunction, microtubule-dependent organelle positioning, ER stress, metabolic intermediates, and post-translational modifications such as palmitoylation. Although these approaches may offer broader metabolic benefits, they generally have lower target specificity and require careful evaluation of off-target effects.

#### 4.3.1 Colchicine

Colchicine inhibits NLRP3 inflammasome activation through multi-stage mechanisms involving P2X7 receptor signaling, K^+^ efflux, and microtubule polymerization. Mechanistic studies suggest that colchicine suppresses P2X7 receptor-mediated K^+^ efflux and disrupts αβ-tubulin polymerization, thereby impairing the microtubule-dependent spatial organization required for inflammasome assembly (140–142). Some studies have also suggested that colchicine may regulate pyrin-related pathways (143).

In metabolic contexts, colchicine has shown early evidence of reducing inflammatory markers and improving insulin resistance in obese adults or patients with metabolic syndrome (144). However, available studies are generally small and short-term, and larger trials are needed to determine whether colchicine provides meaningful metabolic benefit. The FDA approval of low-dose colchicine for cardiovascular risk reduction should not be interpreted as direct approval for obesity, insulin resistance, or T2DM treatment.

#### 4.3.2 Taurine

Taurine is a naturally occurring sulfonic acid derivative present at high intracellular concentrations. Recent experimental evidence suggests that taurine flux may regulate NLRP3 inflammasome activation in macrophages. Exogenous taurine supplementation has been reported to suppress caspase-1 activation, GSDMD cleavage, IL-1β/IL-18 release, and pyroptosis in selected experimental systems, with apparent specificity for NLRP3 over AIM2 or NLRC4 inflammasomes (145).

Beyond inflammasome regulation, taurine has been associated with improved lipid metabolism, reduced serum cholesterol and triglycerides, and improved glucose control in animal and human metabolic studies (146). A meta-analysis of randomized controlled trials in metabolic syndrome reported reductions in systolic blood pressure, diastolic blood pressure, fasting blood glucose, and triglycerides. However, an RCT in patients with T2DM found improvements in insulin and HOMA-IR but not in HbA1c, fasting glucose, or lipid profiles. Most clinical studies are short-term, and the causal relationship between taurine-mediated inflammasome inhibition and metabolic improvement in humans remains indirect (147).

#### 4.3.3 Bax inhibitor-1

Bax inhibitor-1 (BI-1) is a negative regulator of IRE1α and contributes to β-cell stress adaptation under high insulin secretory demand. In diabetic states, BI-1 has been reported to attenuate IRE1α ribonuclease activity and reduce NLRP3 inflammasome-mediated β-cell death, thereby limiting metabolic dysfunction in experimental models (148). These findings suggest that BI-1 may represent a protective regulatory pathway in β-cell stress responses. However, BI-1 is not currently a clinically validated drug target in metabolic disease, and clinical evidence supporting BI-1-directed therapy is unavailable.

#### 4.3.4 Arachidonic acid

Arachidonic acid (AA) is traditionally considered a precursor of pro-inflammatory lipid mediators. However, recent evidence suggests that AA itself may suppress NLRP3 inflammasome activation in selected experimental settings, whereas some downstream metabolites do not show the same inhibitory effect. Mechanistically, AA has been reported to inhibit phospholipase C activity, reduce JNK1 activation, and thereby attenuate NLRP3 inflammasome activation (149).

Nevertheless, the biological effects of AA are complex and context-dependent. A systematic review of randomized trials found that increased AA intake had limited overall effects on lipid profiles, blood pressure, platelet function, coagulation parameters, and inflammatory or immunological markers (150). Therefore, AA should be discussed as a potential endogenous regulator of NLRP3 activity in experimental systems rather than as a clinically established metabolic therapy.

#### 4.3.5 Phenylpyruvic acid-related strategies

Enhanced NLRP3 palmitoylation increases NLRP3 stability by reducing lysosomal degradation, thereby promoting inflammasome activation and IL-1β release. In diabetic foot ulcers, phenylpyruvic acid (PPA) has been reported to accumulate and to be internalized by macrophages through CD36. Internalized PPA binds palmitoyl-protein thioesterase 1 (PPT1), inhibits its depalmitoylase activity, and increases NLRP3 palmitoylation, thereby promoting inflammasome activation (151).

At present, PPA itself is not a conventional therapeutic target, and no approved agents directly targeting PPA are available. Potential future strategies may include reducing PPA biosynthesis through modulation of phenylalanine metabolism, blocking PPA uptake through receptors such as CD36, or restoring PPT1 activity. These approaches remain theoretical and require substantial experimental and clinical validation.

### 4.4 Natural products or multi-target agents

Natural products and traditional medicine-derived compounds have shown extensive mechanistic potential in modulating NLRP3 inflammasome signaling. However, most evidence remains preclinical or is supported by low-level clinical evidence. Major translational challenges include poor bioavailability, uncertain target specificity, lack of standardization, limited reproducibility, small sample sizes, and insufficient long-term safety data. Therefore, these agents should be discussed as candidate modulators rather than established therapeutic options.

#### 4.4.1 Berberine

Berberine is an isoquinoline alkaloid with anti-inflammatory and metabolic regulatory properties. In macrophage-based preclinical studies, berberine suppressed NLRP3 inflammasome activation and reduced IL-1β secretion (152). Separate hepatocyte studies showed that berberine attenuated palmitate-induced inflammatory responses and insulin resistance in HepG2 cells and improved insulin signal transduction partly through reducing endoplasmic reticulum stress (153, 154).

Clinical evidence suggests that berberine may improve glycemic control and obesity-related parameters. A meta-analysis of 37 studies reported significant hypoglycemic effects associated with baseline fasting plasma glucose and HbA1c levels (155). Another systematic review reported reductions in body weight, BMI, and waist circumference, although effects on waist-to-hip ratio were not significant (156). However, many trials are limited by variable study quality, short follow-up, insufficient safety reporting, and lack of standardized formulations. Therefore, berberine should not be presented as a standard inflammasome-targeted therapy for metabolic disease.

#### 4.4.2 Resveratrol

Resveratrol is a naturally occurring polyphenol that suppresses NLRP3 inflammasome activation partly by attenuating mitochondrial dysfunction and reducing IL-1β secretion and macrophage pyroptosis (157). In preclinical T2DM models, resveratrol improves glycemic control by increasing GLUT4 expression in skeletal muscle and GLUT2 expression in liver, thereby enhancing glucose uptake and metabolic homeostasis (158).

A meta-analysis of 30 studies reported that resveratrol supplementation reduced insulin resistance and HbA1c levels, although effects on fasting glucose were significant mainly in diabetic populations (159). Despite these findings, clinical evidence remains heterogeneous, and resveratrol is still best described as an investigational metabolic modulator rather than an established therapy.

#### 4.4.3 Ginsenoside Rb2

Ginsenoside Rb2 is a bioactive saponin derived from *Panax* species. In high-fat diet-induced obese mouse models, ginsenoside Rb2 reduced body weight, adipose tissue accumulation, and inflammatory cytokine levels. Mechanistically, inhibition of adipocyte pyroptosis through downregulation of NLRP3 inflammasome-associated proteins, including caspase-1, ASC, and GSDMD, has been proposed to contribute to improved obesity-related insulin resistance (160, 161). However, poor oral absorption, low bioavailability, and lack of rigorous human trials limit its translational potential.

#### 4.4.4 Salidroside

Salidroside, also known as rhodioloside, is a phenylpropanoid glycoside derived from *Rhodiola rosea*. Experimental studies suggest that salidroside may exert antioxidant and anti-inflammatory effects in diabetes-related complications. In diabetic animal models, salidroside has been reported to suppress NLRP3 inflammasome activation by inhibiting P2X7 receptor expression, thereby ameliorating diabetic neuropathic pain (162). Additional studies suggest that salidroside may improve insulin resistance and diabetic neuropathic pain through AMPK–NLRP3 inflammasome-related pathways (163).

Human studies of *Rhodiola rosea* products have mainly focused on exercise performance and anti-fatigue effects rather than metabolic disease treatment (164). Therefore, salidroside remains at an early stage of clinical translation for metabolic or diabetes-related inflammatory complications.

#### 4.4.5 Brazilin

Brazilin, an isoflavonoid compound isolated from *Caesalpinia sappan* L, has been identified as a natural product inhibitor of the NLRP3 inflammasome. In macrophage and mouse inflammatory models, brazilin suppresses ASC speck formation, caspase-1 activation, and IL-1β release, while human metabolic-disease evidence remains unavailable (165). Therefore, brazilin should be considered a preclinical NLRP3-modulating candidate rather than an established metabolic therapy.

#### 4.4.6 Genipin

Genipin is an iridoid derivative derived from *Gardenia jasminoides* fruit and is the bioactive aglycone of geniposide. Experimental studies suggest that genipin ameliorates high-fat diet-induced obesity and adipocyte pyroptosis partly by inhibiting UCP2-related cell death pathways. It has also been reported to attenuate diet-induced hepatic injury and modulate free fatty acid-induced pyroptotic signaling in metabolic tissues (166).

However, evidence remains largely preclinical, and no robust clinical trials have established genipin as a therapy for obesity-related metabolic disorders.

#### 4.4.7 Huí Yáng Shēng Jī Decoction

HYSJD is a traditional Chinese herbal formulation used for chronic wound healing, including diabetic skin ulcers. Integrative multi-omics analyses combining transcriptomics, network pharmacology, and metabolomics suggest that HYSJD may promote wound healing through modulation of the NF-κB/STAT3/NLRP3 signaling axis. Its active component betaine has been reported to promote macrophage proliferation and M2-like polarization, with increased IL-10 and reduced TNF-α expression (167).

Although HYSJD has shown preliminary efficacy in diabetic wound models, current evidence is limited by small sample size, variable formulation, and non-standardized study design. High-quality randomized clinical trials are needed before it can be considered an evidence-based therapy.

#### 4.4.8 Artesunate

Artesunate is an artemisinin derivative with established clinical use as an antimalarial drug. Experimental studies suggest that artesunate may attenuate diabetic complications by modulating NF-κB/NLRP3 signaling, restoring gut microbiota homeostasis, and reducing oxidative stress and inflammatory responses in diabetic animal models (168).

Although artesunate has a well-characterized safety profile in malaria treatment, evidence supporting its efficacy in metabolic diseases remains limited and largely preclinical. Therefore, its role as an inflammasome-modulating therapy for metabolic disease remains exploratory.

#### 4.4.9 Angoroside C

ANC is a phenylethanoid glycoside identified from *Scrophularia ningpoensis*, a traditional herb used in diabetes-related contexts. LC-MS/MS analysis and molecular docking identified ANC as a candidate AMPK-interacting compound. In db/db mice, ANC activated AMPK, suppressed excessive NLRP3 inflammasome activity, improved glucose and lipid metabolic disturbances, and ameliorated insulin resistance (169).

However, pharmacokinetic studies indicate that the oral bioavailability of ANC in rats is low, approximately 2.1% after oral administration at 100 mg/kg (170). Therefore, despite promising preclinical activity, poor bioavailability and lack of clinical evidence remain major barriers to translation.

#### 4.4.10 Calenduloside E

CE is a triterpenoid saponin reported to modulate NLRP3 inflammasome activation. Experimental evidence suggests that calenduloside E suppresses pyroptosis in macrophages and white adipose tissue by promoting sirtuin 2-mediated deacetylation of NLRP3, especially at lysine 24, thereby attenuating adipose tissue inflammation through the sirtuin 2 –NLRP3 axis (171).

CE has also been reported to exert anti-MASLD effects by targeting pyroptosis-related signaling. RNA-seq enrichment analysis suggested involvement of the PI3K/AKT pathway, and subsequent experiments indicated that CE may inhibit inflammasome-mediated pyroptosis through suppression of PI3K/AKT/NF-κB signaling (172). However, pharmacokinetic analysis suggests very low bioavailability, and clinical evidence is currently unavailable (173).

#### 4.4.11 Benzoylmesaconine

BMA is an alkaloid derived from *Aconitum* species. Experimental studies suggest that BMA inhibits NLRP3 inflammasome activation by attenuating intracellular K^+^ efflux and disrupting inflammasome complex assembly (174). *In vitro*, BMA reduces IL-1β secretion and GSDMD-N expression. *In vivo*, BMA alleviates inflammatory symptoms in MSU-induced acute gout and DSS-induced colitis models.

Although these findings support BMA as a potential anti-inflammatory NLRP3 modulator, its role in metabolic diseases such as obesity, insulin resistance, and MASLD/MASH remains insufficiently explored. Further studies are required to define its metabolic relevance, pharmacokinetics, safety, and clinical applicability.

## 5 Conclusion

The NLR family and its associated inflammasome pathways act as important molecular links between metabolic dysregulation and chronic inflammation in metabolic disorders, including obesity, T2DM, MASLD/MASH, and atherosclerosis. This review summarizes the structural features, activation mechanisms, and disease-specific functions of selected NLR family members, including NLRP3, NLRP1, NLRP6, NLRC5, NLRP12, and NLRP2. Among them, NLRP3 has the strongest evidence base in metabolic inflammation, whereas NLRP1, NLRP6, NLRC5, NLRP12, and NLRP2 show more context-dependent roles with variable levels of support. In particular, NLRC5-related metabolic effects should be interpreted mainly through transcriptional regulation, antigen presentation, inflammatory signaling, and lipid-metabolic associations, while NLRP12/PANoptosis-related mechanisms and NLRP2-mediated metabolic regulation remain emerging or exploratory areas.

Therapeutic strategies targeting NLR inflammasome pathways include direct inflammasome/NLR inhibitors, downstream cytokine blockade, indirect modulators of inflammasome activation, and natural or multi-target bioactive compounds. These approaches may attenuate metabolic inflammation through inhibition of inflammasome assembly, blockade of IL-1β/IL-18-related signaling, modulation of upstream metabolic stress pathways, and regulation of gut microbiota or tissue inflammatory microenvironments. However, clinical translation remains challenging because many candidate compounds are still supported mainly by cell or animal studies, and only selected anti-inflammatory therapies have been tested in large randomized clinical settings. Future studies should clarify cell-type-specific NLR functions, distinguish inflammasome-dependent from inflammasome-independent mechanisms, and validate candidate pathways in human metabolic disease to support safer and more precise inflammasome-targeted interventions.

## Glossary

NLR: NOD-like receptor
PAMPs: pathogen-associated molecular patterns
DAMPs: damage-associated molecular patterns
MAMPs: metabolism-associated molecular patterns
T2DM: type 2 diabetes mellitus
T1DM: type 1 diabetes mellitus
MASLD: metabolic dysfunction-associated steatotic liver disease
MASH: metabolic dysfunction-associated steatohepatitis
NAFLD: non-alcoholic fatty liver disease
NASH: non-alcoholic steatohepatitis
MAFLD: metabolic dysfunction-associated fatty liver disease
IL-1β: interleukin-1β
IL-18: interleukin-18
IL-17: interleukin-17
Th17: T helper 17 cells
LPS: lipopolysaccharide
ATP: adenosine triphosphate
TLRs: Toll-like receptors
TLR4: Toll-like receptor 4
CLRs: C-type lectin receptors
NF-κB: nuclear factor kappa-light-chain-enhancer of activated B cells
MAPK: mitogen-activated protein kinase
IFN: interferon
NOD: nucleotide-binding oligomerization domain
NOD1: nucleotide-binding oligomerization domain-containing protein 1
NOD2: nucleotide-binding oligomerization domain-containing protein 2
RIPK2: receptor-interacting serine/threonine-protein kinase 2
LRR: leucine-rich repeat
NACHT: NAIP, CIITA, HET-E, and TP1
CARD: caspase recruitment domain
PYD: pyrin domain
NLRA: NLR family member with acidic transactivation domain
NLRB: NLR family member with BIR domain
NLRC: NLR family member with CARD domain
NLRX: NLR family member with unknown effector domain
NLRP: NLR family member with PYD domain
ASC: apoptosis-associated speck-like protein containing a CARD
AIM2: absent in melanoma 2
GSDMD: gasdermin D
NEK7: NIMA-related kinase 7
RIPKs: receptor-interacting serine/threonine kinases
HCK: hematopoietic cell kinase
NAD+: nicotinamide adenine dinucleotide
ROS: reactive oxygen species
KAT5: lysine acetyltransferase 5
PHGDH: phosphoglycerate dehydrogenase
FFAs: free fatty acids
MyD88: myeloid differentiation primary response 88
SPARC: secreted protein acidic and rich in cysteine
Lcn2: lipocalin-2
TET2: ten-eleven translocation 2
DPP9: dipeptidyl peptidase 9
CARD8: caspase recruitment domain-containing protein 8
AMPK: AMP-activated protein kinase
MHC-I: major histocompatibility complex class I
LTA: lipoteichoic acid
BMI: body mass index
HDL: high-density lipoprotein
SNPs: single-nucleotide polymorphisms
PPARγ: peroxisome proliferator-activated receptor gamma
TGF-β: transforming growth factor beta
ERK: extracellular signal-regulated kinase
IRAK1: interleukin-1 receptor-associated kinase 1
TRAF3: TNF receptor-associated factor 3
NLRP12-AID: NLRP12-associated autoinflammatory disease
SCFAs: short-chain fatty acids
GPCRs: G protein-coupled receptors
Nrf2: NF-E2-related factor 2
HFD: high-fat diet
CAPS: cryopyrin-associated periodic syndromes
MSU: monosodium urate
hsCRP: high-sensitivity C-reactive protein
HOMA-IR: homeostatic model assessment of insulin resistance
BMDMs: bone marrow-derived macrophages
AA: arachidonic acid
BI-1: Bax inhibitor-1
PPA: phenylpyruvic acid
PPT1: palmitoyl-protein thioesterase 1
HYSJD: Huí Yáng Shēng Jī Decoction
ANC: angoroside C
CE: calenduloside E
BMA: benzoylmesaconine
PI3K: phosphoinositide 3-kinase
AKT: protein kinase B
UCP2: uncoupling protein 2
STAT3: signal transducer and activator of transcription 3
